# Predictive and Prognostic Value of Non-Coding RNA in Breast Cancer

**DOI:** 10.3390/cancers14122952

**Published:** 2022-06-15

**Authors:** Navid Sobhani, Richard Chahwan, Raheleh Roudi, Rachel Morris, Stefano Volinia, Dafei Chai, Alberto D’Angelo, Daniele Generali

**Affiliations:** 1 Section of Epidemiology and Population Sciences, Department of Medicine, Baylor College of Medicine, Houston, TX 77030, USA; dafei.chai@bcm.edu; 2 Institute of Experimental Immunology, University of Zurich, CH-8057 Zurich, Switzerland; chahwan@immunology.uzh.ch; 3 Molecular Imaging Program at Stanford, Department of Radiology, Stanford University, Stanford, CA 94305, USA; roudi@stanford.edu; 4 Thunder Biotech, 395 Cougar Blvd, Provo, UT 84604, USA; rmorris@thunderbiotech.com; 5 Department of Morphology, Embryology and Medical Oncology, Università Degli Studi di Ferrara, 44100 Ferrara, Italy; stefano.volinia@osumc.edu; 6 Department of Biology & Biochemistry, University of Bath, Bath BA27AY, UK; ada43@bath.ac.uk; 7 Department of Medical Surgery and Health Sciences, University of Trieste, 34127 Trieste, Italy; dgenerali@units.it

**Keywords:** non-coding RNA, biomarkers, breast cancer, prognostic, diagnostic

## Abstract

**Simple Summary:**

In our journey to fight cancer, biomarkers for prediction and efficacy of therapies could help medical practitioners to give patients the best available treatments in a timely fashion. Non-coding RNA, in most recent times, has been shown to play crucial roles for cancer growth. An exponential increase of evidence in the clinical literature supports their implication to predict patients’ outcomes. In this article, we summarize the current predictive and prognostic value of non-coding RNA in breast cancer.

**Abstract:**

For decades since the central dogma, cancer biology research has been focusing on the involvement of genes encoding proteins. It has been not until more recent times that a new molecular class has been discovered, named non-coding RNA (ncRNA), which has been shown to play crucial roles in shaping the activity of cells. An extraordinary number of studies has shown that ncRNAs represent an extensive and prevalent group of RNAs, including both oncogenic or tumor suppressive molecules. Henceforth, various clinical trials involving ncRNAs as extraordinary biomarkers or therapies have started to emerge. In this review, we will focus on the prognostic and diagnostic role of ncRNAs for breast cancer.

## 1. Introduction

**Figure 1:**
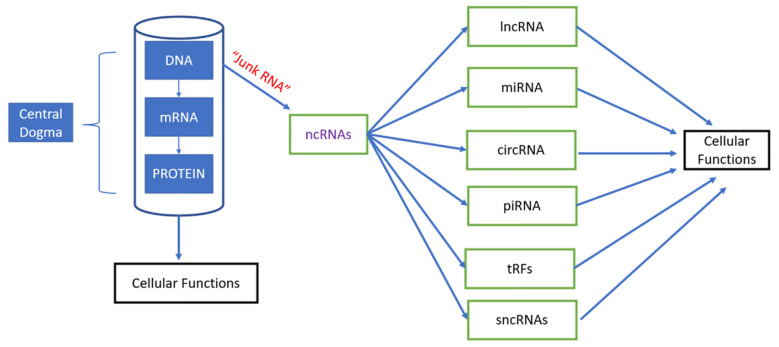
From the Central Dogma to ncRNAs production in regulating cellular functions. Originally, it had been postulated as the “central dogma” that there is a flow of information that is passed from DNA in the nucleus, transcribed into messenger RNA (mRNA) which travels outside the nucleus of the cells into the cytoplasm where it is translated into proteins. Proteins are the building blocks of cells and are crucial for cellular functions. The DNA that was transcribed into non-coding RNA (ncRNA) was considered as ”junk RNA”. However, in the past decades this concept has been revolutionized with the discovery of the critical roles of ncRNAs (such as long non-coding RNA [lncRNA], microRNA [miRNA], circulatory RNA [circRNA], PIWI-interacting RNA [piRNA], tRNA-derived fragments [tRFs], small ncRNA [sncRNAs]) in regulating cellular functions and their implications in medical conditions, such as breast cancer.

From the moment Francis Crick’s central dogma asserted that genetic information travels from DNA through RNA towards protein synthesis [1,2], a lot of research has shown that RNAs are not only intermediary copies of genetic information (mRNA), components of the ribosome (ribosomal RNAs, rRNAs), or translators of codon sequence (tRNAs) [3]. The total percentage of genes encoding proteins account only for the 2% of the genome and the remaining had been considered as “junk” non-coding RNA before being discovered to have critical roles in cellular biology, such as silencing genes, after the first discovery of small RNAs, lineage defective 4 (lin-4) [4] and lethal 7 (let-7) [5] in C elegans about 23 and 16 years, respectively. Since then a multitude of non-coding RNAs (ncRNA) species have been described, some of which are highly conserved, such as microRNAs (miRNAs), transcribed regions that are ultra-conserved [6], and circular RNAs (cricRNAs) and other regions that are not conserved among species, such as long ncRNAs (lncRNAs) [7] and tRNA-derived fragments (tRFs), which may modulate the immune response and act as miRNAs [8,9] (Figure 1). The miRNA are made of about 22 nucleotides (nt), are the most common type of ncRNAs that are studied and control translation of mRNA into proteins by silencing genes [10,11]. On average, miRNAs regulate the translation of more than 60% of protein-coding genes. The piRNAs are another type of ncRNAs that are about 24–30 nt in length, which are Dicer-independent and bind the PIWI subfamily of Argonaute family proteins that are involved in maintaining genome stability in germline cells [12,13]. The snoRNAs are an intermediate-sized ncRNAs of about 60–300 bp. The lncRNAs are a heterogenous group of ncRNAs of about 200 nt, involved in many biological processes. They constitute the largest portion of ncRNAs [14]. There are other types of ncRNAs that are associated with transcriptional start sites (TSSs) of genes that are associated with human diseases [15].

**Figure 2:**
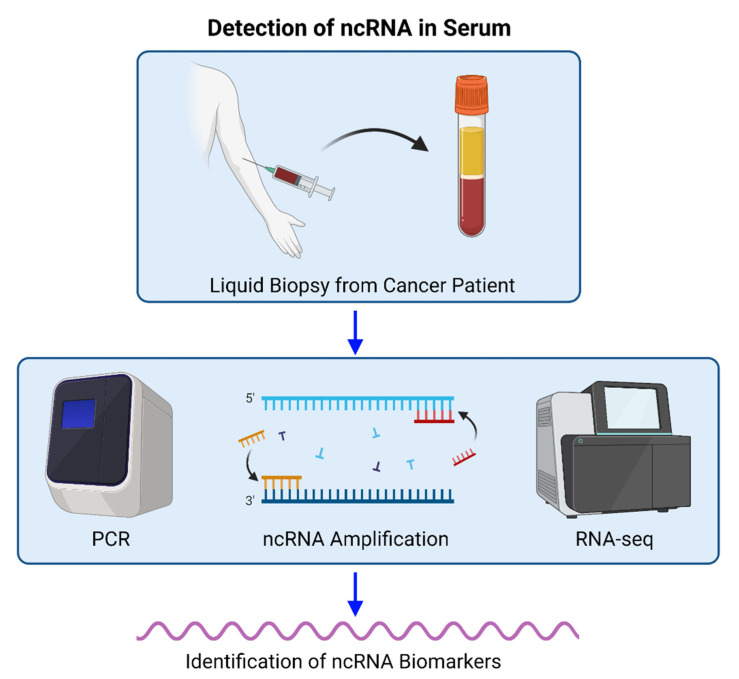
Circulating non-coding RNA biomarkers discovery for diagnostic or prognostic prediction of BC. Blood of patients is processed to obtain serum, non-coding RNAs are amplified through PCR (either quantitative real-time PCR or digital droplet PCR). Biomarkers for prediction are discovered based on their correlation with patients’ survival or response to therapy.

Most fascinatingly in oncology is that some ncRNAs, such as miRNAs, are capable of targeting the mRNAs of many other different genes involving cancer. Additionally, miRNAs are able to interact functionally with other ncRNAs species to regulate stability of circRNAs and lncRNAs. In turn, the lncRNAs and circRNAs regulate the abundance of miRNAs that are available through sequestration [16]. In this review we will focus on the predictive and prognostic values of non-coding RNAs in breast cancer patients from clinical investigations in the literature (Figure 2).

## 2. Long Non-Coding RNA Molecular Mechanisms and Cancer Involvement

The mammalian genome is represented by a limited number of protein-coding genes. The Encyclopedia of DNA Elements (ENCODE) project estimates that nearly 75% of the human transcriptome is dominated by non-coding transcripts that are highly present in non-ribosomal non-mitochondrial RNAs [17,18]. Studies indicate that approximately 80% of non-coding RNAs are related to functional DNA sequences within the human genome and that some non-coding RNAs may have important functions, such as facilitating communication between mitochondria and other cellular compartments, despite not coding for protein [19,20]. Compared to protein-coding sequences, less is understood about non-coding RNAs. Long non-coding RNAs (lncRNAs) have some characteristics in common with mRNAs. For example, lncRNAs are generally transcribed by RNA polymerase II (Pol II) and have a 5′-end 7-methyl guanosine (m^7^G) cap and 3′-end polyadenylated tail [21]. However, further study has revealed unique differences between lncRNA and mRNA transcription, localization, processing, gene regulation, export into the cytosol, and involvement in cancer.

### 2.1. LncRNA Nuclear Accumulation, Splicing, and Gene Regulation

High concentrations of lncRNA are localized in the nucleus due to transcription and processing mechanisms that differ from mRNA [22,23]. Transcription stages may be altered by phosphorylation of the carboxy-terminal domain on Pol II, which dysregulates Pol II and promotes lncRNAs that have weak co-transcriptional splicing, polyadenylation-independent termination, and rapid degradation by the exosome after accumulating on chromatin [24]. Chromatin-tethered lncRNAs, that have multiple U1 small nuclear RNA binding sites for recruiting U1 small nuclear ribonucleoprotein (U1snRNP) to Pol II, can escape targeted nuclear surveillance. As a result, more non-coding RNAs become tethered to chromatin [25]. Additional lncRNAs gather on chromatin when the Pol II-associated elongation factor SPT6 function is lost [26]. SPT6 is required for chromatin transcription, Pol II is released from the paused stage to initiate elongation and acts as a histone chaperone [27]. Nojima et al. revealed that SPT6 depletion results in H3K36me3 histone mark redistribution from protein-coding genes to lncRNA genes, which enhances lncRNA transcription. Further investigation found that SPT6 knockdown inhibits Integrator complex recruitment, which normally terminates lncRNA transcription, leading to increased chromatin-restricted lncRNAs that can form RNA:DNA hybrid (R-loops) and potentially induce DNA damage [26]. These studies suggest that lncRNAs dominate the nucleus in contrast to mRNAs, which are mainly cytosolic [23]. A recent study indicated that cytoplasmic accumulation region (CAR-N) removal in NKLA, an intron-less lncRNA, results in significant nuclear restriction and insertion of CAR-N increased cDNA transcript export. Researchers also found that TREX-TAP pathway depletion strongly restricts NKILA to the nucleus and that removal of CAR-N does not prevent breast cancer cell migration [28]. Previously, Zuckerman et al. depleted several proteins that are involved in nuclear export. The results suggested that TREX and NXF1 depletion promoted lncRNA nuclear retention, TREX depletion affected G/C-rich and spliced transcripts, and NXF1 affected the export of transcripts with high A/U-rich regions or containing few but long exons [29].

Generally, lncRNAs are characterized as having long distances between the branch point and 3′ splice site, weaker splicing signals, and a short polypyrimidine tract (PPT) [21]. In fact, one study indicated that lncRNAs are sometimes not properly spliced and 3′ end processed due to splicing inefficiency, which contrasts with mRNA splicing efficiency [24]. Some lncRNAs may regulate splicing, recently, Li et al. discovered that lncRNA DKFZp434J0226 promotes phosphorylation of splicing factor SF3B6, which regulates pre-mRNA alternative splicing and promotes pancreatic ductal adenocarcinoma (PDAC) carcinogenesis [30]. Non-coding RNAs are also known to alter gene expression and disease progression by binding protein complexes. LncRNAs are thought to directly influence gene regulation because they usually remain attached to their site of transcription and recruit proteins [31,32]. One well-studied lncRNA is the X inactive-specific transcript (Xist), which is necessary to transcriptionally silence one X-chromosome in female development [33]. Xist transcription yields a 15–17 kb long lncRNA that remains tethered to the X-chromosome and recruits complexes to repress X-linked genes in *cis* [34]. Sequence motifs in *cis* may influence lncRNA localization. For example, RNA-binding proteins (RBPs) can recognize *cis*-regulatory elements on RNA to further guide RNA cellular localization [35]. RBP peptidylprolyl isomerase E (PIPIE) can be differentially expressed to promote nuclear retention by inhibiting lncRNA splicing [21]. Splicing factor expression may also contribute to lncRNA accumulation in the nucleus. Guo et al. showed that PIPIE inhibits lncRNA splicing and significantly increases nuclear localization in mouse embryonic stem cells (mESCs) [22]. Another study reported that HNRNPK, a nuclear RBP, could increase nuclear accumulation of both lncRNAs and mRNAs by binding to C-rich motifs [36]. C-rich motifs and nuclear retention element U1 snRNA-binding site recruit U1 snRNP to increase nuclear retention [37]. Additional investigation revealed that short sequences from Alu elements could also be bound by HNRNPK and enhance nuclear accumulation [36]. In conclusion, multiple transcription and processing factors coordinately control lncRNA nuclear localization and influence gene regulation.

### 2.2. Export and Cellular Compartmentalization of LncRNA

**Figure 3:**
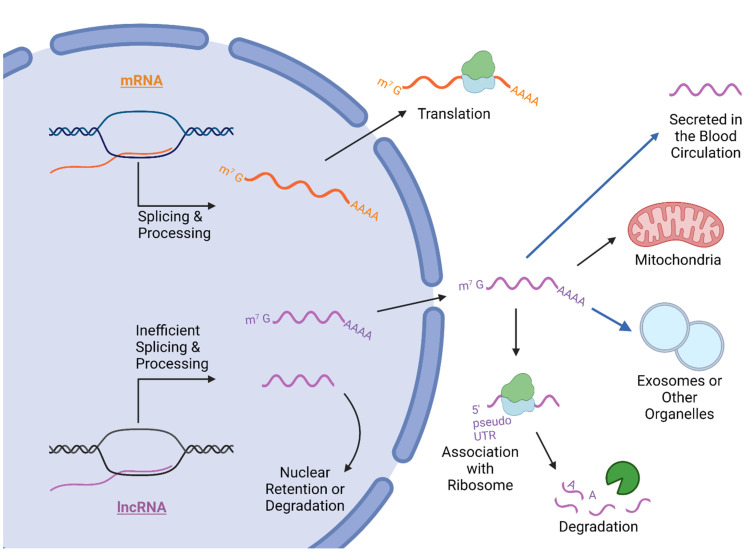
Biogenesis of lncRNA. LncRNA is either restricted to the nucleus, degraded, or exported from the nucleus into the cytoplasm. Outside the nucleus, lncRNA may localize to the mitochondria, other organelles, associate with ribosomes, or be secreted into the blood circulation with or without exosomes.

LncRNAs may be exported to the cytosol and localized to various cellular compartments. Some lncRNAs are even detected in human blood exosomes most likely after being bound by RBPs at specific sequences [38,39]. Some exosomal lncRNAs can even mediate tumor microenvironment communication [40]. For example, exosomal lncRNA urothelial cancer-associated 1 (UCA1) enhances CD133+ cervical cancer cell differentiation and self-renewal via micro-rRNA-122-5P/SOX2 axis and UCA1 silencing leads to inhibited cell proliferation and invasion [41]. DOCK9-as2 is another exosomal lncRNA that decreases papillary thyroid carcinoma cell migration, epithelial-mesenchymal transition (EMT), and invasion when it is down-regulated [42]. A recent study used ribosome profiling techniques to identify sequence features that were related to ribosomal association of mouse and human lncRNAs [43]. Ji et al. performed bioinformatics to determine that some lncRNAs have pseudo-5′UTRs that may be translated into functional proteins [44]. LncRNAs that are bound to ribosomes are reported to regulate translation and are eventually degraded [45,46,47]. The mechanism for ribosomal association of lncRNA is still unknown and the number of ribosome-associated lncRNAs is limited; therefore, ribosomal lncRNA remains an area for further investigation. In addition to ribosomes, lncRNAs are also exported to mitochondria from the nucleus and produced from the mitochondrial genome directly [48,49]. Human antigen R (HuR) mediates lncRNA ribonuclease mitochondrial RNA processing gene (RMRP) localization from the nucleus to the mitochondria. After RMRP arrives to the mitochondria, G-rich RNA sequence-binding factor 1 (GRSF1) binds RMRP and helps it move to the matrix. GRSF1 depletion lowers RMRP levels and reduces oxygen consumption and mitochondrial DNA replication [50]. LncRNAs may regulate mitochondrial metabolic reprogramming, mitophagy, and mitophagy-associated drug resistance [40]. Metastasis-associated lung adenocarcinoma transcript 1 (MALAT1) is found in high amounts in hepatocellular carcinoma cell (HCC) mitochondria. Zhao et al. showed that altered CpG methylation of mitochondrial DNA in HCC cells decreased oxidative phosphorylation, ATP production, mitophagy, and increased mitochondrial apoptosis. Further study indicated that HuR and mitochondrial carrier homolog 2 (MTCH2) facilitated MALAT1 localization to mitochondria [51]. lncRNA export suggested that lncRNA is involved in important functions throughout the body. Additionally, small ncRNA are 18–200 nucleotides long, constitute a large family of endogenously expressed transcripts also playing crucial roles in regulating cell function [52,53]. While their exact function is unknown, various studies suggest they might be involved in gene expression regulation at the level of post-transcriptional mRNA processing [54,55] and ribosome biogenesis [56]. Figure 3 illustrates lncRNA biogenesis, compartmentalization, or exportation of from the cell into the blood circulation.

### 2.3. LncRNA in Cancer Processes and Patient Serum

Recent studies have shown that lncRNAs may be associated with cancer proliferation, survival, EMT, and metastasis [57,58]. In contrast, some lncRNAs can also promote metastatic dormancy. Recently, Liu et al. showed that lncRNA NAS1 upregulation in mesenchymal-like breast cancer stem-like cells (BCSCs) increases both metastatic dormancy and cancer cell dissemination. Additional study revealed that lncRNA NR2F1-AS1 (NAS1) binding to GC-rich 5′UTR region of NR2F1 mRNA recruits PTBP1 to increase NR2F1 translation, which suppresses Δ*Np63* expression and promotes EMT alterations and impaired tumorigenicity [59]. Another study indicated that NAS1 expression was associated with late recurrence of estrogen receptor (ER)-positive breast cancer. Researchers identified NAS1 as a potential biomarker for ER+ breast cancer and determined that lncRNA was related to hormone receptor expression [60]. Kim et al. showed that inactivated MALAT1 promotes lung metastasis in a transgenic mouse model and is reversed when MALAT1 is restored. However, MALAT1 overexpression inhibited breast cancer metastasis in a mouse model. MALAT1 expression normally prevents TEAD, a pro-metastatic transcription factor, from binding coactivator YAP and other gene promoters. Knockout of MALAT1 in human breast cancer cells also promoted metastasis and was reversed by re-adding MALAT1 expression [61]. In summary, lncRNAs (e.g., HOTAIR, CARMN, and LINC00478) may regulate cancer processes in multiple ways and be used as cancer biomarkers. Specifically, the clinical significance of lncRNA detection in the blood is becoming increasingly important in identifying potential biomarkers and therapeutic targets in patients with cancers, such as breast cancer. Recently, quantitative real-time PCR (qRT-PCR), digital droplet PCR (ddPCR), and RNA sequencing (RNA seq) have been used to investigate ncRNA potential biomarkers for breast cancer in many studies. These studies will be summarized in the next main section of this review and Table 1.

## 3. Clinical Value of Non-Coding RNAs in BC

The clinical value of 6-lncRNAs was assessed by Zhang et al. in HER2+ BC patients’ data from the Cancer Genome Atlas (TCGA). The authors formulated an RNA network to explore the role of these core lncRNAs in HER2+ BCs from 113 HER2+ BC and 105 tumor-adjacent normal breast tissues. The high expression of four lncRNAs (AC046168.1, AC010595.1, AC069277.1, and AP000904.1) was significantly correlated with worse OS, while the expression of MIR762MG and LINC00528 was associated with worse OS. The 6-lncRNA model had a good predictive power for OS (*p* < 0.0001) and three-year survival (AUC = 0.980) in HER2+ BCs [63].

Du et al. investigated the prognostic value of miR-92b as a biomarker of disease prognosis in 112 female BC patients with qRT-PCR treated in their hospital compared to 108 healthy women. They observed a remarkably higher expression level of miR-92b-3p (*p* < 0.05), AUC of 0.88. The expression was correlated with degree of differentiation, tumor size and TNM staging, lymph metastasis (*p* < 0.05). In addition, they showed that miR-92b significantly positively correlated with the expression of carbohydrate antigen 125 (CA125) (*p* < 0.05). They concluded that miR-92b-3p is important biomarker for diagnose and evaluate BC [64]. Li et al. investigated the prognostic value of four immune-related lncRNAs in TNBC through data of patients from TCGA and GEO databases. From a total of 62 immune-related lncRNAs, they discovered the following four lncRNAs with independent prognostic values: RP11-1024P17.1, RP11-890B15.3, MFI2-AS1 and RP11-180N14.1. These four lncRNAs could stratify patients into high and low risk groups, with low risk having unfavorable outcomes. Furthermore, they observed that RP11-890B15.3, RP11-180N14.1 and RP11-1024P17.1 could regulate more mRNAs by targeting various miRNAs. The MF12-AS1 regulated three mRNAs by sponging miR-3150a-3p. These four lncrNAs could be used as prognostic biomarkers and targeting them could have therapeutic benefits, as also suggested by the authors [64]. Ye et al. investigated the lncRNA MIAT biomarker in 1057 BC and 103 healthy patients specimens first with TCGA and then they confirmed it with qRT-PCR. Their expression could be used as a prognostic indicator of mortality risk in BC patients in different aspects. The expression of MIAT in serum positively correlated with TNM stage (*p* = 0.032) and lymph node metastasis (*p* = 0.028) [65]. Said et al. investigated miR-222 capacity to inhibit tumor suppressor CDK inhibitor p27 in 110 BC patients with qRT-PCR. As expected, they observed that miR-222 was expressed at significantly higher levels in BC. Consequently, in BC patients the levels of CDK inhibitor p27 was lower than the control group. Serum p27 and miR-222 could help differentiate between BC and controls [66]. Zhu et al. investigated the prognostic value of lncRNA signatures using first TCGA and then validating the relative expression levels of eight lncRNA using qRT-PCR in 808 BC patients. The risk scores were determined in the following way: genes with hazard ratio (HR) values that were less than 1 represented protective genes, whereas genes with HR values that were higher than 1 represented increased-risk genes. Based on the expression levels that were observed, patients with low-risk scores had higher OS than those with high-risk scores. They concluded that their 8-lncRNA panel could be a potential prognostic biomarker for BC [67]. Wu et al. investigated the prognostic value of autophagy-related lncRNAs in BC. They explored the prognostic value of this interesting lncRNAs, namely the LINC01871, MAPT-AS1 and AL122010.1, AC090912.1, and AC061992.1. Their model offered an independent prognostic value (HR = 1664, 1381–2006), with a risk score that was significantly related to the TNM stage, PR, ER, and HER2 status in BC patients [68] (it is ref PMID: 33694315). Su et al. studied the lncRNA-protein coding gene (CG) relationship and lncRA-PCG co-expression information in correlation with BC. Their bio-informatics analysis was able to identify 30 risk lncRNAs for BC, and was able to distinguish normal and tumor samples. Through a gene ontology analysis, they further showed that risk lncRNAs mainly synergistically exerted functions that were related to cell cycle and DNA separation and replication. They developed a 4-lncRNA prognostic signature, made of LINC01977, AP000851.1, MAFG-DT, and SIAH2-AS1 to assess survival accuracy of the signature by performing time-dependent receiver operating characteristic (ROC) analysis. The area under the curve (AUC) of survival prediction for the ROC curve for 1, 3, 5, and 10 years were 0.68, 0.61, 0.62, and 0.63, respectively. The 4-lncRNA signature was an independent prognostic marker for BC [69]. Sheng et al. investigated the prognostic value lncRNA CARMN gene as a down-regulated gene by miR143-3p in triple negative breast cancer (TNBC), a hard-to treat subtype of breast cancer lacking effective treatment targets with usually a very poor prognosis. Besides looking at the expression of CARMN, the authors explored the downstream expression of CARMN with RNA-sequencing (RNA-seq) too. They were able to determine that CAMN could predict both better prognosis and higher response rate of cisplatin-based neoadjuvant chemotherapy in BC patients. The RNA-seq data further revealed that CARMN could inhibit DNA replication. An important DNA replication initiation factor is MCM5, which is the most down-regulated gene in the DNA replication pathway following CARMN overexpression. The CARMN could produce miR143-3p from exon 5, which is DROSHA- and DICER-dependent, resulting in binding and a decrease of MCM5. Suppressing the miR143-3p could weaken the CARMN function in order to suppress tumorigenesis in the promotion of chemosensitivity [70]. Zhao et al. examined the role of miR-25-3p as a biomarker in BC with qRT-PCR. The research group detected miR-25-3p expression levels in tissues and serum samples from various BCs to evaluate their prognostic value. Further cellular function assays, dual-luciferase reporter assays, and Western blotting were used to discover the miR-25-3p targets. miR-25-3p was up-regulated in the BC and serum samples compared to normal BC and serum samples. The patients with high serum miR-25-3p levels were more likely to develop lymph node metastasis vs. patients with low serum levels of miR-25-3p. The AUC for miR-25-3p for the diagnosis of BC was 0.748, with a 57.1% sensitivity and a 95.0% specificity. The knockdown of miR-25-3p suppressed BC proliferation and invasion. In addition, transducer of ERBB2 1 (TOB1) was identified as the primary potential target of miR-25-3p. Overall, patients with low expression levels of serum miR-25-3p had a higher survival compared to those with higher miR-25-3p expression. Therefore, miR-25-3p could be a good biomarker for BC [71]. Kärkkäinen et al. sought to resolve the debate on whether PIWI-interacting RNAs (piRNAs) could serve as a potential biomarker in BC. They conducted small RNA sequencing in 227 fresh-frozen BC samples using qRT-PCR from the Eastern Finnish Kuopio BC project to study the presence of piRNAs in BC and their association with clinicopathological features and outcome of BC patients. They observed that the presence of three small piRNAs, namely the DQ570994, DQ571955, and DQ596932 in the samples were up-regulated in Grade III tumors and DQ696932 was up-regulated in estrogen receptor negative tumors only. Additionally, patients with estrogen-positive (ER+) tumors and higher DQ571955 had shorter relapse-free survival and poorer BC-specific survival. Such findings indicated that DQ571955 could be a candidate predictive biomarker for radiotherapy response in ER+ BCs. DQ570994 was a candidate predictive marker for tamoxifen and chemotherapy response. Overall, these three small RNAs have their own values as biomarkers for BC [72]. Haug et al. investigated the prognostic value of the mRNA-miRNA-lncRNA network in TNBC using the GEO2R tool. They used the Enrichr and STRING to conduct a protein-protein interaction and pathway enrichment analysis, respectively. Through an upstream analysis of lncRNAs and miRNAs they could identify miRNet and mirTarBase, respectively. They examined the prognostic values and determined that from 860 upregulated and 622 down-regulated mRNAs, 10 key miRNAs upstream of this key hub genes could have a predictive value. Of these, six up-regulated miRNAs (hsa-let-7e-5p, hsa-miR-19a-3p, hsa-miR-130b-3p, hsa-miR-18b-5p, hsa-miR-98-5p, and hsa-miR-222-3p) were significantly associated with poor prognosis and four down-regulated miRNAs (hsa- let-7b-5p, hsa-miR-10b-3p, hsa-let-7a-5p, and hsa-miR- 410-3p) were associated with good prognosis in TNBC. A total of two key lncRNAs (NEAT1 and MAL2) were shown to be able to differentiate good prognosis vs. bad regression-free survivals (*p* < 0.05) [73]. Sharma et al. investigated the prognostic value of lncRNA ZFAS1 in TNBC. They found that the expression of the ZFAS1 was significantly down-regulated (about three-fold) in the blood samples of TNBC patients (*n* = 40) compared to matched healthy controls (*n* = 40). Through functional analysis they observed that ZFAS1 promoted the proliferation of human breast cancer cell line MDA-MB-231 TNBC cells through the inhibition of the cyclin-dependent kinase (CDK) inhibitors p21 and p27. The down-regulation of ZFA1 led to a decrease in the protein levels of epithelial markers E-cadherin, Claudin-1, and Zo-1, while increasing the levels of mesenchymal markers Slug and ZEB1. They identified a strong negative correlation between ZFAS1 and signal transducer and activator of transcription 3 (STAT3) gene expression (*p* = 0.0002). Also, they observed that a decrease in the level of ZFAS1 (*p* < 0.05) significantly correlated with increased STAT3 and phosphorylated STAT3 at Ser727 residue in TNBC. Overall, they observed that ZFAS1 could be a diagnostic and prognostic marker for TNBC that could be also used for therapy [74]. Bajaj et al. investigated the prognostic value of miR-182 and miR-18 a in 50 locally advanced TNBC using qRT-PCR. A significant correlation was observed with clinical nodal status, T-category, clinical response, pathological response with miR-18, and miR-182 (*p* < 0.005) [75]. Huang et al. conducted a meta-analysis, collecting data from 933 patients from 11 articles. The results revealed that low miR-153 expression significantly correlated with poor OS (HR = 2.45, 95%; CI = 1.66–3.63, *p* < 0.001), but not with disease-free survival (DFS). Overall, from this study they showed that miR-153 could be a very effective biomarker for tumor prognosis especially in BC and digestive tumors [76]. Xiong et al. investigated the prognosis and diagnostic values of miR-196a in cancers, including BC. They searched though cancer-related databases and their pooled results indicated that miR-196a was a valuable diagnostic biomarker in cancer (AUC 0.87, 95% CI:0.84–0.90; sensitivity (SEN) = 0.73, 95% CI:0.64–0.81; specificity (SPE) = 0.90, 95% CI: 0.81–0.95). Moreover, the pooled analysis revealed that elevated miR-196a levels in tumor tissues (HR = 2.54, 95% CI: 1.79–3.61, P_Heterogeneity_ = 0.000, I^2^ = 75.8%) or serum and plasma (HR = 4.06, 95% CI: 2.67–6.18, P_Heterogeneity_ = 0.668, I^2^ = 0%) correlated with unfavorable survival. In conclusion, tumor tissue or blood-derived miR-196a could be used a prognostic and diagnostic biomarker for cancers such as BC [77]. Another meta-analysis reported that miR-206 plays an important role in cancers and it could be used as a prognostic biomarker. The pooled HR showed that low miR-206 expression was significantly associated with unfavorable OS (HR = 2:03, 95 CI%: 1.53–2.70, *p* < 0:01). Moreover, the expression of miR-206 predicted significantly negative association with tumor stage (III–IV vs. I–II) (OR = 4:20, 95% CI: 2.17–8.13, *p* < 0:01), distant metastasis, lymph node status (OR = 3:58, 95%: 1.51–8.44, *p* = 0:004) and invasion depth (T3 + T4 vs. T2 + T1) (OR = 2:43, 95%: 1.70–3.49, *p* < 0:01) [78]. Gao et al. showed that the overexpression of miR-1225 promoted BC progression resulting in a poor prognosis in 120 BC patients. The authors looked at the miR-1225 expression and observed that it was significantly up-regulated in BC and associated with TNM stage of BC. The JAK1 was identified as a direct target of miR-1225, which was involved in cell proliferation, invasion, migration, and invasion of BC. Overall, the overexpression of miR-1225 in BC could be used as a biomarker since it correlated with a poor prognosis of patients and promoted the progression of BC by targeting JAK1 [79]. Wang et al. investigated the prognostic value of tissue differentiation-inducing non-protein coding RNA (TINCR) in the pathogenesis of various human tumors such as BC. They used qRT-PCR to detect serum lncRNA TINCR levels in 72 TNBC patients, 105 non-TNBC patients, 60 benign BC, and 86 heathy subjects. They showed that the lncRNA TINCR level was significantly increased in BC patients, particularly in TNBC. The clinicopathological features and clinical outcomes of TNBC were worse in patients with high circulating lncRNA TINCR [80]. Luo et al. investigated endogenous RNA network to discover new lncRNA, miRNA, and mRNA biomarkers with diagnostic and prognostic value for early BC based on TCGA data 787 early BC patients and 78 normal individuals. Furthermore, they validated their findings by qRT-PCR. They discovered six biomarkers: ADAMTS9-AS1, CDKN2B-AS1, IL-6, MMP11, hsa-miR-145-5p, and hsa-miR-182-5p, whose AUC were 0.947, 0.862, 0.842, 0.993, 0.960, and 0.944, and the specificity and sensitivity 83.4% and 95.6%, 72.2%, and 90.3%, 80.1%, and 74.3%, 96.2%, and 96.5%, 90.1%, and 92.3%, and 88.7% and 90.4%, respectively [81]. The prognostic value of lncRNA TPT1-AS1 was investigated by Hu et al. in breast cancer firstly via TCGA and GEO, secondly through qRT-PCR. They observed that TPT1-AS1 was significantly correlated with clinical features of malignancy such as shorter OS, lymph node metastasis, TNM stage, and Ger-2 status. They concluded that TPT1-AS1 could be used as an independent prognostic factor in BC patients [75].

Zhang et al. investigated the prognostic value of exosomal miR-1246 and miR-155 as predictive and prognostic biomarkers for transtuzumab-based therapy resistance in HER2+ BC. Firstly, they isolated miRNA from exosomes of four transtuzumab-resistant and four trastuzumab-sensitive patients, who were profiled through miRNA microarray. Secondly, they validated the predictive and prognostic roles of the filtered miRNAs in 107 early-stage and 68 metastatic BC patients that were treated with trastuzumab-based chemotherapy. The most upregulated miRNAs in trastuzumab-resistant HER2+ BC patients were miR-1246 and miR-155, which were further validated in the trastuzumab-resistant patients’ samples (*n* = 32) vs. trastuzumab-sensitive cases (*n* = 36). The miR-1246 showed an ROC curve of 0.750 with 78.1% sensitivity and 75% specificity in discriminating resistant from sensitive patients (*p* < 0.001), whereas miR-155 showed an ROC curve area of 0.877 with 68.8% sensitivity and 97.2% specificity (*p* < 0.001). However, both miRNAs were not associated with OS. The study showed that miR-146 and miR-155 could distinguish trastuzumab-resistant from -sensitive patients [82]. Sun et al. explored 9-lncRNA signature to predict the distant relapse-free survival of 10,231 HER2- BC patients receiving taxane and anthracycline-based neoadjuvant chemotherapy. They built a scoring system in which the higher the score the lower the survival probability. The ROC AUC was 0.777 to 0.823 from one- to seven-year survival rate. The model and it individual lncRNAs were able to differentiate the survival probability between higher scores and lower scores. The prognostic power was comparable to that of the PAM50 signature. The study indicated that the 9-lncRNA signature was a robust and effective model for the prediction of DRFS of patients with HER2- BC [83]. Lei et al. investigated the prognostic value of miR-20b in BC by qRT-PCR looking into 123 BC patients and the correlation with clinical pathological features. They observed that the expression of miR-20b increased with an increase in tumor grade (*p* < 0.05). Additionally, high expression levels of miR-20b correlated with stromal overgrowth, high stromal atypia and cellularity, infiltrative tumor margin, tumor grade, high mitotic activity, local recurrence, and metastasis (*p* < 0.05) The high expression of miR-20b correlated with shorter DFS (*p* < 0.001). Overall, the study suggested that miR-20b could be used as a prognostic biomarker in BC [84]. Wang et al. studied the value of exosomal miR-148a as a new biomarker for the prognosis of BC. They used qRT-PCR to evaluate the expression of miR-148a in BC patients with benign tumors and healthy controls. The serum levels of exosomal miR-148a were gradually down-regulated from the healthy control patients with benign breast tumors to BC patients. The serum exosomal miR-148a levels could distinguish the BC patients from the healthy volunteers. A significant correlation was observed between serum exosomal miR-148a and tumor-node-metastasis (TNM) stage, lymph node metastasis, and differentiation. Additionally, the lower expression of serum exosomal miR-148a in patients corelated with worse OS and DFS than patients with higher serum miR-148a exosomal levels. The study evinced that serum exosomal miR-148a is significantly reduced in BC patients and the down-regulation of serum exosomal miR-148a was closely associated with an unfavorable clinical outcome of BC [85]. Li et al. studied the prognostic and diagnostic potential of miR-1204 in BC. They studied the miR-1204 expression in tissue and serum samples by qRT-PCR in 144 BC patients and 38 healthy volunteers. They observed that both in the tissues and serum samples that miR-1204 was an independent prognostic factor. The study suggested that miR-1204 could be used a prognostic and diagnostic biomarker for BC [86].

Zhang et al. conducted a meta-analysis to study the clinicopathological and prognostic value of lncRNA SNHG6 in cancers, including in BC. The systematic search showed that in 914 patients from 13 studies, the SNHG6 expression could predict unfavorable OS (HR = 2.04, 95% CI: 1.56–2.52). Moreover, the elevated level of SNHG6 was positively associated with tumor invasion depth (OR = 1.76, 95% CI: 1.18–2.63), DM (OR = 1.90, 95% CI: 1.37–2.64), LNM (OR = 1.60, 95% CI: 1.18–2.17), and advanced TNM stage (OR = 1.88, 95% CI: 1.36–2.60) [87]. Binabaj conducted another meta-analysis for the prognostic investigation of miR-21 in cancer patients. They investigated 10,213 BC patients and 76 studies. Through subgroup analysis, high miR-21 correlated with shorter OS in breast cancer patients (HR = 2.20; 95% CI: 1.78–2.73; *p* = 0.001). Therefore, the miR-21 could be a clinically useful biomarker for cancer progression [88]. Zhang et al. investigated the prognostic potential of lncRNAs in BC by looking at the RNA expression levels through TCGA. The diagnostic value of all differentially expressed lncRNAs were studied with the ROC curve. Core lncRNA-mRNA co-expression networks on the basis of weighted gene co-expression network (WGCNA) were constructed and functional enrichment analysis run using Database of Annotation, Visualization and Integrated Discovery (DAVID). The diagnostic value was further evaluated by an independent dataset from GEO. They revealed that seven core lncRNAs, namely LINC00478, AL035610.1, AC005550.4, MIR143HG, MIR497HG, PGM5-AS1, and RP11-175K6.1 had a good single-factor diagnostic value for BC. Moreover, AC005550.2 had a prognostic value for BC. AC005550.4 and MIR497HG were able to distinguish better BC patients in early stage from patients at advanced stages. Overall, the 7-lncRNA could have a prognostic value in BC [89]. Feng et al. investigated the prognostic value of has-mir-124 in predicting the outcome of BC based on bioinformatic analysis. They conducted an analysis of cancer genome atlas for breast invasive carcinoma (TCGA_BRCA) for has-mir-124. They reported that the OS of patients with high expression levels of has-mir-124-1 and has-mir-124-2 was better than that of patients with low expression levels of has-mir-124-1 and has-mir-124-2. The expression of has-mir-124-1 and has-mir-124-2, has-mir-124-3 was mainly enriched in T1/T2 stages, M0 stages, and N0/N1 stages. There was a negative association between has-mir-124-1 and has-mir-124-2, has-mir-124-3 and lymph node metastasis. Overall, has-mir-124 was associated with worse clinical outcomes and could be used as a biomarker for BRCA [90].

Li et al. investigated the prognostic value of miR-1179 in 164 BC. They studied through qRT-PCR the expression levels of miR-1179 in both BC tissues and cell lines to find any association between its level of expression with clinicopathological features and patients’ prognosis. They observed that miR-1179 was frequently down-regulated in BC tissues and cell lines. Also, that low miR-1179 levels correlated with advances clinical stage, shorter OS, and lymph node metastasis. Moreover, fain-of-function assays indicated that overexpression of miR-1179 was able to significantly suppress BC proliferation, migration, and invasion through the inhibition of the expression of Notch1, Notch2, and Hes1. Overall, miR-1179 could serve as a new prognostic biomarker or actionable target for new therapies [91]. Zhao et al. conducted a meta-analysis to study the prognostic significance of lncRNA in BC antiestrogen resistance 4 (BCAR4). The meta-analysis included 1293 patients from 9 studies. They observed that BCAR4 expression was significantly correlated with poor OS (HR = 1.98, CI: [1.71–2.29]), *p* < 0.00001, and high expression levels of BCAR4 correlated with worse clinical stage, distant metastases, and lymph node metastasis [92]. A meta-analysis that was conducted by Geo et al. looked into the prognostic value of miR-93 in various solid cancers, including BC. The authors observed that the AUC for overall sensitivity and specificity were 0.76 (0.64–0.85), 0.82 (0.64–0.92), and 0.85 (0.82–0.88), suggesting that miR-93 is a good prognostic biomarker. They further validated the prognostic value of miR-93 with qRT-PCR and saw that the serum levels of this miRNA were higher in breast cancer and breast hyperplasia. Overall, the authors concluded that miR-93 is a good prognostic biomarker for survival prediction in cancers such as BC [93]. Cheng et al. studied the ability of 3-miRNA signature to predict clinical outcomes in 1103 BC patients vs. 104 healthy samples. Their study first identified 106 differentially expressed miRNAs in BRCA tissues and matched normal tissues, including 81 up-regulated miRNAs and 25 down-regulated miRNAs. They established a set of 3-miRNA signature (miR-133a-2, miR-204, and miR-301b) that was significantly up-regulated in BRCA patients to classify patients into high-risk and low-risk groups. Conclusively, the 3-miRNA signature could be a potential biomarker for BRCA [94]. A meta-analysis of Cai et al. confirmed the prognostic potential for miR-2031-3p in BC. They detected that miR-2031-3p was markedly upregulated in 1077 BC tissues compared to 104 adjacent BC samples from TCGA. Additionally, they observed that miR-2031-3p was expressed in 756 BC tissues vs. 76 adjacent BC tissues using samples from the University of California Santa Cruz Xena project. A comprehensive meta-analysis finally showed that the University of California Santa Cruz Xena project was markedly expressed in 2444 BC samples compared to 559 adjacent breast tissues. Both ROC and sROC revealed that their expression could distinguish between BC and adjacent breast tissues. The study showed that insulin-like growth factor receptor (IGF1) was a hub gene that was associated with miR-2031-3p. However, the miR-2031-3p did not have prognostic value in BC. Overall, the study suggests that miR-2031-3p could enhance tumorigenesis in BC, but has no prognostic utility [95]. Kawaguchi et al. investigated the role of miRNA for the prediction of metastasis and prognosis in BC. They used TCGA data to look into the prognostic value of miRNAs in 1051 BC patients. A total of three miRNAs risk score (miR-19a, miR-93, and miR-106a) was developed through this TCGA cohort that was capable of independently predicting poor prognosis (*p* = 0.0005) of known clinical risk factors. They validated their data using additional three independent cohorts: the GSE22220 (*p* = 0.0003), GSE19536 (*p* = 0.0009), and the Molecular Taxonomy of Breast Cancer International Consortium (*p* = 0.0023). Interestingly this 3-miRNAs risk score was able to predict bone recurrence in TCGA (*p* = 0.0052). This finding was further validated in another independent population of patients experiencing bone recurrence. Overall, the miRNA-based risk score could predict worse survival and bone recurrence in BC patients [96]. Cui et al. investigated the prognostic significance of lncRNA maternally expressed gene 3 (MEG3) in BC through bioinformatics analysis. The MEG3 was more frequently down-regulated in BC than in normal tissues. On the contrary ER and progesterone receptor (PR) status positively correlated with MEG3 expression. The TNBC status and the Scarff Bloom and Richardson grade criterion negatively correlated with MEG3 expression. MEG3 positively correlated with heparin sulfate proteoglycan 2 (HSPG2) expression in BC. Overall, MEG3 could be a good predictor of prognosis of BC with HSPG2 [97]. A competing endogenous RNA network by Fan et al. identified four lncRNA-miRNA-mRNA signatures to predict prognosis in 1103 BC vs. 104 adjacent normal breast tissues. The authors obtained RNA sequencing data and clinical characteristics of BC patients from the TCGA. A total of 2150 DEmRNAs, 1061 DElncRNAs, and 82 DEmiRNAs were identified between BC and normal breast tissues. An lncRNA-miRNA-mRNA network of BC was established that was made of 48 DElncRNA, 10 DEmRNAs, and 8 DEmiRNAs. Through a univariate regression analysis of the DElncRNAs, 7 lncRNAs (LINC00536, LINC00491ADAMTS9-AS1, AC061992.1, AL391421.1, HOTAIR, and TLR8-AS1) were associated with OS of BC patients. Furthermore, four of those lncRNA had a prognostic value. The cumulative risk score indicated that the 4-lncRNA signature could independently predict OS in BC patients. The AUC of the 4-lncRNA signature that was associated with three-year of survival was 0.696. Overall, the 4-lncRNA is a good prognostic tool for BC [98]. Fan et al. investigated the prognostic value of 3-lncRNAs as diagnostic and prognostic biomarkers of TNBC. They implemented a comprehensive analysis of lncRNA expression from the clinical data of 1097 BC samples from the TCGA. Overall, 1510 differentially expressed lncRNAs in normal and TNBC samples were extracted. On the same note, 672 differentially expressed lncRNAs between nTNBC and TNBC samples were detected. The AUC revealed that three upregulated lncRNAs, namely AC091043.1, AP000924.1, and FOXCUT may have a strong diagnostic value for the prediction of TNBC in both training and validation sets (AUC > 0.85). Moreover three other lncRNAs, namely AC010343.3, AL354793.1, and FGF10-AS1, were associated with TNBC prognosis (*p* < 0.05). Multivariate Cox regression analysis suggested that this 3-lncRNA signature was a good independent prognostic factor of clinical variables predicting OS in TNBC and was able to classify patients into high- and low- risk subgroups. Overall, the data that were presented by the research group suggests that the 3-lncRNA signature could be efficient in predicting diagnosis and prognosis of TNBC [99]. Zhang et al. conducted a meta-analysis to question the prognostic value of lncRNA TUBA4B in various cancers, including BC. They showed that TUBA4B was significantly correlated with OS (HR = 1.33, 95% CI: 1.16–1.52, *p* = 0.000), DFS (HR = 1.25, 95% CI: 1.06–1.48, *p* = 0.007), and recurrence-free survival (RFS; HR = 1.42, 95% CI: 1.26–1.60, *p* = 0.000). Additionally, TUBA4B was a risk factor for BC (HR = 1.52, 95% CI: 1.10–2.12, *p* = 0.012). Overall, the study suggested that low levels of TUBA4B are significantly associated with short OS, DFS, and RFS in cancers and that TUBA4B could therefore be a BC biomarker [100]. Tang et al. investigated the prognostic value of a new miRNA-based signature in BC patients. They used a TCGA database and their preliminary candidates were screened out using a univariate Cox regression test. They then used a LASSO Cox regression analysis to select the following biomarkers as predictors of OS: hsa-let-7b, hsa-mir-9-3, hsa-mir-22, hsa-mir-30a, hsa-mir-31, hsa-mir-101-2, hsa-mir- 135a-2, hsa-mir-320b-1, hsa-mir-493, hsa-mir-556, hsa-mir-652, hsa-mir-874, hsa-mir-3130-1, hsa-mir-3678, hsa-mir-4662a, hsa-mir-4772 and hsa-mir-6733. On the other hand, sa-mir-130a, hsa-mir-204, hsa-mir-217, hsa-mir-223, hsa-mir-24-2, hsa-mir-29b- 1, hsa-mir-363, hsa-mir-5001, hsa-mir-514a-1, hsa-mir-624, hsa-mir-639, hsa-mir-659, and hsa-mir-6892 were a predictive signature for RFS. The ROC analysis validated the accuracy and stability of these two signatures. Through functional analysis, the genes that were targeted by the miRNAs were enriched in the following pathways: MAPK/RTK, Ras, and PI3K-Akt signaling pathways. To summarize, the study identified two novel miRNA-based signatures functioning as independent prognostic indicators for BC patients, which deserves prospective studies validation [101]. Xu et al. examined the prognostic value of circRNA OMA1 in the regulation of BC. They used qRT-PCR in 64 pairs of BC tissues and adjacent normal tissues. They observed that the expression levels of circOMA1 were upregulated in the BC tissues and associated with tumor size and lymph node metastasis. Additionally, the expression of circOMA1 could discriminate between BC tissues and adjacent normal tissues. On the functional level, circOMA1 promoted viability, migration, and invasion of BC cells. On the other hand, knocking down circOMA1 had the opposite effect. circOMA1 promoted tumor progression by up-regulating sirtuin 4 (SIR4) expression and sponging miR-1276. Overall, circOMA1 together with mir-1276/SirT4 could be prognostic markers for BC [102]. Dwedar et al. investigated the prognostic value of cell-free circmiRNAs miR-10b and its target soluble E-cadherin in tissue specimens for diagnosis and prognosis. They observed that in 61 BC patients and 48 healthy volunteers the serum levels of miRNA10b, assessed by qRT-PCR, and E-cadherin expression level in serum, assessed by ELISA technique, was significantly upregulated in BC patients compared to the controls. Moreover, the expression levels of serum miR-10b was progressively up-regulated in advanced stages of BC with higher levels in metastatic vs. non-metastatic BC. The combination of both serum miR-10b and sE-cadherin showed greater sensitivity and specificity in detecting BC metastasis (02.9% and 97.9%, respectively) with an AUC of 0.98, 95% CI (0.958–1.00). Overall, the study evinced that simultaneous detections of miRNA10b and E-cadherin could be a robust serum biomarker to determine diagnosis and prognosticate BC metastasis [103]. Zheng et al. investigated the clinical significance of lncRNA SPRY4-IT1 in predicting ethe fficacy and survival of 93 BC patients undergoing neoadjuvant chemotherapy. SPRY4-IT1 was detected by q-RT-PCR. The bioinformatic analysis from this study indicated that SPRY4-IT1 correlated with chemo-resistance in BC. SPRY4-IT1 was significantly expressed at higher levels in cancer tissues, such as BC, than normal tissues (*p* < 0.05) and its higher expression also correlated with higher rates of lymph node metastasis (*p* = 0.002) and recurrence (*p* = 0.017). High expression of SPRY4-IT1 was also indicative of poor clinical response in the whole group, luminal A subgroup and luminal B subgroup (*p* < 0.05), and pathological complete response in the whole group. Overall, the study showed that SPRY4-IT1 expression was a prognostic biomarker of poor clinical response in BC [104]. Lasham et al. investigated the predictive role of cell-free ncRNA in 30 BC and 10 healthy individuals. They quantified ncRNAs with ddPCR and discovered miR-923. Furthermore, they quantified CA 15-3 protein levels in the samples. The miR-923 and CA 15-3 at BC surgery time were significantly associated with prognosis, independent from treatment (*p* = 3.9 × 10^−3^ and 1.9 × 10^−9^, respectively). Overall, the study showed that a combination of miR-923 and CA 15-3 detected from serum are a good preoperative, noninvasive prognostic marker of BC [105]. Nama et al. enquired on the prognostic validity of miR-138 in TNBC. The level of miR-138 increased significantly in TNBC and it correlated with poor prognosis in the patients and functionally relevant cancer progression. Therefore, the authors suggested that the miR-138 was a good diagnostic and prognostic biomarker of TNBC [106]. Zou et al. conducted a meta-analysis of the prognostic value of ncRNA plasmacytoma variant translocation 1 (PVT1) in 2095 solid tumors, including BC. Their study included 39 articles and 3974 patients. They discovered that PVT1 correlated with OS of cancers (HR = 1.6, 95% CI: 1.50–1.78, *p* < 0.000001). The PVT1 value correlated also with low OS in BC. Additionally, PVT1 positively correlated with TNM stage, tumor size, lymph node metastasis, and distant metastasis [107]. Tang et al. explored the relationship between serum exosomal HOTAIR with poor OS to neoadjuvant chemotherapy and response to tamoxifen therapy in 15 BC patients that were treated surgically and 15 healthy individuals that were enrolled as controls; 25 BC patients received neoadjuvant chemotherapy before surgery and another 25 BC patients received tamoxifen hormone treatment after surgery patients. The exosomes were isolated from serum, tumor tissues, and cell culture medium. After confirming exosomes with electron microscopy and Western blotting, qRT-PCR was conducted to assess HOTAIR expression. The serum exosomal HOTAIR level was significantly higher in BC patients than healthy individuals (*p* < 0.001). Moreover, the HOTAIR levels at three months after surgery was markedly decreased compared to the levels before surgery (*p* < 0.001) and expression levels of HOTAIR in cell culture medium was higher in the BC cell lines. The AUC for serum exosomal HOTAIR was of 0.9178 with CI of 95% CI of 0.8407–1.017 (*p* < 0.01). The AUC of CA15-3 was 0.7378 (95% CI, 0.5585–0.9170, *p* = 0.03). The higher the expression of exosomal HOTAIR, the worse the DFS (*p* = 0.0481) and OS (*p* = 0.0463). Overall, the study suggested that serum HOTAIR could be a diagnostic and prognostic biomarker, since its high expression correlated with poor response to neoadjuvant chemotherapy and tamoxifen response [108]. Lukianova et al. enquired on the prognostic value of miRNAs in 28 HER2+ BC. They ran IHC tissue and sera samples with q-RT-PCR to look at the relative expression levels of miR-155, miR-320a, and miR-205. Circulating expression of miR-155, miR-320a, and miR-205 correlated with lymph node metastases and basal breast cancer subtype. In conclusion, their report indicated that miR-155, miR-320a, and miR-205 could provide just some information on the major clinical-pathological features of BC, but not be used as a biomarker [109]. The prognostic significance of miR-34 rs4938723 T>C polymorphism has been investigated in TNBC by Andriani et al. through IHC. They analyzed data from 114 TNBC and blood samples from 124 healthy donors. In ductal BC patients (69.4%), the TC or CC genotype were common (*p* = 0.020). Patients with TC or CC alleles had a higher survival (*p* < 0.001). TC or CC or single nucleotide polymorphism correlated with shortened DFS in female patients (*p* = 0.05). In TNBC the TC/CC genotype exhibited shorter OS (*p* < 0.001). In women with the TC or CC genotype together with ductal histology had a significantly shorter survival (*p* < 0.001). Overall, the data from this paper suggest that TC and CC alleles are associated with unfavorable prognosis in TNBC and that they could be used as a prognostic biomarker [110]. Pan et al. computationally investigated the RNA-seq database for prognostic miRNAs for BC. The authors built a novel computational approach called ProModule to analyze prognostic biomarkers using this module perspective. The ProModule works in two main stages: (1) first it finds individual miRNA biomarkers using univariate and multivariable Cox proportional hazards regressions; (2) second it employs a clustering method to systematically detect miRNA-mediated modules using prognostic statistical significance [111]. Fan et al. conducted an in silico study and identified a series of dysregulated microRNAs in TNBC that were able to provide better diagnosis and predict OS in 1098 TNBC from TCGA. In total, 289 miRNAs were aberrantly regulated in TNBC tissues compared to adjacent, non-cancerous tissues in TNBC compared to non-TNBC. They discovered the following four miRNAs with diagnostic value (AUC > 0.8): hsa-miR-10a, hsa-miR-18a, hsa-miR-135b, and hsa-miR-577. In summary, the multivariate Cox-s proportional hazards regression model indicated that the 4-miRNA signature was an independent prognostic factor of clinical variables in TNBC patients. Therefore, the 4-miRNA signature could be used as a prognostic biomarker for TNBC patients [112].

Wang et al. investigated the prognostic value of five lncRNA in BC. They screened over the TCGA though the core of competitive endogenous RNAs and found that the following miRNAs constituted a signature with a great potential to predict OS in BC patients at different stages though competitive binding to miR-10b: ACTA2-AS1, RP11-384P7.7, RP11-327J17.9, RP11-124N14.3, and RP11-645C24.5. Overall, these five ncRNAs could be used as biomarkers to predict potential diagnosis and prognosis of BC [113]. The prognostic potential value of circmiR-122 has been uncovered by Saleh et al. The authors investigated miR-122 in 90 BC and 60 healthy controls with qRT-PCR. They also detected CA15-3 and carcinoembryonic antigen levels with ELISA. They observed that the levels of miR-122 in BC were higher than in controls and the higher miR-122 levels were detected in patients with metastasis. In addition, the paper showed that miR-122, at a cut-off value > 2.2, had a sensitivity of 93.3% and a specificity of 90% in its capacity to distinguish BC from controls and it could predict metastasis at a cutoff value > 10.9 with a sensitivity of 95.83% and a specificity of 65.15%. In conclusion, the study showed that miR-122 expression could be a biomarker for OS and PFS [114]. Arabkari et al. analyzed the relative expression value of miRNAs that are associated with luminal A BC. They used RT-qPCR to look at the expression levels of 10 miRNAs in the whole blood samples from luminal A from 38 luminal A BC patients vs. 20 healthy controls. Of the 10 miRNAs, the absolute RT-qPCR method identified six miRNAs that were upregulated (miR-16, miR-145, miR-155, miR- 451a, miR-21, and miR-486) and one that was down-regulated miRNA (miR-195) in BC. The best diagnostic value for the luminal A BC was obtained for three miRNAs (miR-145, miR-195, and miR-486), with an AUC of 0.875, a sensitivity of 76%, and a specificity of 81%. In conclusion, the authors suggested the absolute value of the three miRNAs had a prognostic value in luminal A BC [115]. Meng et al. conducted a meta-analysis for the prognostic value of lncRNA BCAR4 in 890 BC. They observed that high lncRNA BCAR4 expression correlated with poor OS (HR 2.80, 95% CI: 2.08–3.78; *p* < 0.001). Moreover, the higher levels of lncRNA BCAR4 significantly correlated with increased tumor stage, lymph node, and distant metastases [116]. Li et al. investigated the prognostic value of miR-1179 in 161 BC patients. They studied with qRT-PCR the expression levels of miR-1179 in both BC tissues and cell lines to find any association between its level of expression with clinicopathological features and patients’ prognosis. They observed that miR-1179 was frequently down-regulated in BC tissues and cell lines. They also observed that low miR-1179 levels correlated with advances clinical stage, shorter OS, and lymph node metastasis. Moreover, fain-of-function assays indicated that the overexpression of miR-1179 was capable to significantly suppress BC proliferation, migration, and invasion through the inhibition of the expression of Notch1, Notch2, and Hes1. Overall, miR-1179 could serve as a new prognostic biomarker or actionable target for new therapies [91]. Zhao et al. conducted a meta-analysis to study the prognostic significance of lncRNA in BC antiestrogen resistance 4 (BCAR4). The meta-analysis included 1293 patients from 9 studies. They observed that BCAR4 expression was significantly correlated with poor OS (HR = 1.98, CI: [1.71–2.29]), *p* < 0.00001, and high expression levels of BCAR4 correlated with worse clinical stage, distant metastases, and lymph node metastasis [92]. A meta-analysis that was conducted by Geo et al. looked into the prognostic value of miR-93 in 491 cancer patients including BC and 391 healthy people. The authors observed that the AUC for overall sensitivity and specificity were 0.76 (0.64–0.85), 0.82 (0.64–0.92), and 0.85 (0.82–0.88), suggesting that miR-93 is a good prognostic biomarker. They further validated the prognostic value of miR-93 with qRT-PCR and saw that the serum levels of this miRNA were higher in breast cancer and breast hyperplasia. Overall, the authors concluded that miR-93 is a good prognostic biomarker for survival prediction in cancers such as BC [93]. Cheng et al. studied the ability of 3-miRNA signature to predict clinical outcomes in 1103 BC from TCGA. Their study first identified 106 differentially expressed miRNAs in BRCA tissues and matched normal tissues, including 81 up-regulated miRNAs and 25 down-regulated miRNAs. They established a set of 3-miRNA signature (miR-133a-2, miR-204, and miR-301b) that was significantly upregulated in BC patients to classify patients into high-risk and low-risk groups. Conclusively the 3-miRNA signature could be a potential biomarker for BC [94]. A meta-analysis of Cai et al. confirmed the prognostic potential for miR-2031-3p in BC. They detected that miR-2031-3p was markedly upregulated in 1077 BC tissues compared to 104 adjacent BC samples from TCGA. Additionally, they observed that miR-2031-3p was expressed in 756 BC tissues vs. 76 adjacent BC tissues using samples from the University of California Santa Cruz Xena project. A comprehensive meta-analysis finally showed that the University of California Santa Cruz Xena project was markedly expressed in 2444 BC samples compared to 559 adjacent breast tissues. Both ROC and sROC revealed that their expression could distinguish between BC and adjacent breast tissues. The study showed that insulin-like growth factor receptor (IGF1) was a hub gene that was associated with miR-2031-3p. However, the miR-2031-3p did not have prognostic value in BC. Overall, the study suggests that miR-2031-3p could enhance tumorigenesis in BC, but has no prognostic utility [95]. Kawaguchi et al. investigated the role of miRNA for the prediction of metastasis and prognosis in BC. They used TCGA data to look into the prognostic value of miRNAs in 1051 BC patients. A total of three miRNAs risk scores (miR-19a, miR-93, and miR-106a) were developed through this TCGA cohort capable to independently predict poor prognosis (*p* = 0.0005) of known clinical risk factors. They validated their data using additional three independent cohorts: the GSE22220 (*p* = 0.0003), GSE19536 (*p* = 0.0009), and the Molecular Taxonomy of Breast Cancer International Consortium (*p* = 0.0023). Interestingly this 3-miRNAs risk score was able to predict bone recurrence in TCGA (*p* = 0.0052). This finding was further validated in another independent population of patients experiencing bone recurrence. Overall, the miRNA-based risk score could predict worse survival and bone recurrence in BC patients [96]. Cui et al. investigated the prognostic significance of lncRNA maternally-expressed gene 3 (MEG3) in BC through bioinformatics analysis. The MEG3 was more frequently down-regulated in BC than in normal tissues. On the contrary, ER and progesterone receptor (PR) status positively correlated with MEG3 expression. The TNBC status and Scarff Bloom and Richardson grade criterion negatively correlated with MEG3 expression. MEG3 positively correlated with heparin sulfate proteoglycan 2 (HSPG2) expression in BC. Overall, MEG3 could be a good predictor of prognosis of BC with HSPG2 [97]. A competing endogenous RNA network by Fan et al. identified four lncRNA-miRNA-mRNA signatures to predict in 155 TNBC prognosis. The authors obtained RNA sequencing data and clinical characteristics of BC patients from the TCGA. A total of 2150 DEmRNAs, 1061 DElncRNAs, and 82 DEmiRNAs were identified between BC and normal breast tissues. An lncRNA-miRNA-mRNA network of BC was established that was made of 48 DElncRNA, 10 DEmRNAs, and 8 DEmiRNAs. Through a univariate regression analysis of the DElncRNAs, 7 lncRNAs (LINC00536, LINC00491ADAMTS9-AS1, AC061992.1, AL391421.1, HOTAIR, and TLR8-AS1) were associated with OS of BC patients. Furthermore, four of those lncRNA had a prognostic value. The cumulative risk score indicated that the 4-lncRNA signature could independently predict OS in BC patients. The AUC of the 4-lncRNA signature that was associated with three-year of survival was 0.696. Overall, the 4-lncRNA is a good prognostic tool for BC [98]. Fan et al. investigated the prognostic value of 3-lncRNAs as diagnostic and prognostic biomarkers of TNBC. They implemented a comprehensive analysis of lncRNA expression from the clinical data of 1097 BC samples from the TCGA. Overall, 1510 differentially expressed lncRNAs in normal and TNBC samples were extracted. On the same note, 672 differentially expressed lncRNAs between nTNBC and TNBC samples were detected. The AUC revealed that three up-regulated lncRNAs, namely AC091043.1, AP000924.1, and FOXCUT, may have a strong diagnostic value for the prediction of TNBC in both training and validation sets (AUC > 0.85). Moreover three other lncRNAs, namely AC010343.3, AL354793.1, and FGF10-AS1, were associated with TNBC prognosis (*p* < 0.05). Multivariate Cox regression analysis suggested that this 3-lncRNA signature was a good independent prognostic factor of clinical variables for predicting OS in TNBC that was able to classify patients into high- and low- risk subgroups. Overall, the data that were presented by the research group suggests that the 3-lncRNA signature could be efficient in predicting diagnosis and prognosis of TNBC [99]. Zhang et al. conducted a meta-analysis to question the prognostic value of lncRNA TUBA4B in various cancers, including 88 BC. The data were verified in 94 cancers. They showed that TUBA4B was significantly correlated with OS (HR = 1.33, 95% CI: 1.16-1.52, *p* = 0.000), DFS (HR = 1.25, 95% CI: 1.06–1.48, *p* = 0.007), and recurrence-free survival (RFS; HR = 1.42, 95% CI: 1.26–1.60, *p* = 0.000). Additionally, TUBA4B was a risk factor for BC (HR = 1.52, 95% CI: 1.10–2.12, *p* = 0.012). Overall, the study suggested that low levels of TUBA4B are significantly associated with short OS, DFS, and RFS in cancers and that TUBA4B could, therefore, be a BC biomarker [100]. The prognostic role of lncRNA RAB6C-AS1 has been investigated by Salavaty et al. in various cancers including breast cancer through qRT-PCR. They showed that RAB6C-AS1 expression is higher in cancers including breast cancer, suggesting it could be used as prognostic marker. However, more data are required to further prove this point [117]. 

Papadopoulos investigated the prognostic value of miR-331 in 130 malignant and 66 benign breast cancers that were surgically-resected from primary tumors using qRT-PCR. They observed that miR-331 significantly correlated with malignant breast tumors compared to their benign counterparts. Therefore, miR-331 could be considered a good prognostic marker for BC [118]. Kim et al. investigated the prognostic value of five microRNAs (miR-134, miR-125b-5P, miRNA-30a, miR-10a-5p, and miR-222) in HR-positive BC during tamoxifen treatment in 176 tumor tissues from HR+ patients receiving tamoxifen through qRT-PCR. These five miRNAs were upregulated in distant recurrence cases within five years. Moreover, they were expressed at significantly higher levels that correlated with short relapse-free time (*p* < 0.0001). Through three miRNAs expression model, a high-risk subset of patients could be categorized with short relapse-free survival (AUC = 0.891, *p*-value < 0.0001). Overall, these five miRNAs could be used to predict distant recurrence during tamoxifen treatment [119]. Jiang et al. studied the correlation between the overexpression of lncRNA LINC01296 with unfavorable BC in 55 paired BC patients with healthy tissues. LINC01296 was observed as overexpressed in several malignancies. LINC01296 was found to be upregulated in larger tumors, advanced TNM stage, and positive lymph node metastasis in BC. Moreover, LINC01296 was an independent prognostic marker for BC. On the mechanistic level, the down-regulation of LINC01296 significantly inhibited BC tumor growth, while enhancing apoptosis, both in vitro and in vivo. Overall, this study indicated that LINC01296 could be a negative prognostic biomarker that could be used to predict disease progression as well as an actionable target [120]. Liu et al. studied the clinical potential of miR-940 as both a diagnostic and prognostic biomarker in BC patients. The authors measured the miR-940 levels with qRT-PCR in BC patients vs. healthy controls (*p* < 0.001). miR-940 was correlated with TNM stage and lymph node metastasis. The AUC was 0.905, with a sensitivity and specificity that ranged of 94.5% and 78.6%, respectively. miR-940 could be an independent prognostic factor (HR = 2.645, 95% CI = 1.426–4.906 and *p* = 0.002). Overall, the paper suggested that miR-940 could be a reliable biomarker for diagnosis and prognosis in 128 BC patients [121]. The lncRNA HOTAIR prognostic value was investigated in correlation with response to neoadjuvant chemotherapy in BC patients. The authors checked HOTAIR levels in the blood of 112 BC patients before neoadjuvant chemotherapy with qRT-PCR. They then looked at the correlation between HOTAIR and clinicopathologic status and response to neoadjuvant chemotherapy. Overall, the high expression levels of HOTAIR correlated with response to neoadjuvant chemotherapy as well as to a worse BC prognosis [122]. Wang et al. investigated the role of miR-330-3p as a prognostic indicator of poor prognosis in BC. The authors used qRT-PCR to check the expression of miR-330-3p in BC tissues vs. normal tissues. The expression of miR-330-3p was significantly higher in 233 BC specimens than the corresponding non-cancerous tissues (*p* < 0.01). Additionally, the miR-330-3p level was positively corelated with lymph node metastasis and TNM stage. The five-year OS of BC with miR-330-3p expression was significantly shorter in patients with low miR-330-3p expression (*p* < 0.0001). Overall, the paper suggests that miR-330-3p upregulation is associated with prognosis in BC patients, suggesting that it could be a prognostic biomarker and an actionable treatment [123]. An integrative bioinformatics approach was used to study ncRNA variants that were associated with BC profiles and outcome by Györiffy et al. through the TCGA database in 930 BC patients. The authors observed that the overall mutation rate in the coding and non-coding regions were significantly higher in ER−/HER2+ tumors (*p* = 0.0028 and *p* = 2.4 × 10^−7^, respectively) [124]. 

In a study that was conducted by Chen et al., the miRNAs that were associated with lymph node metastasis and prognosis in 449 BC patients were explored [125]. They identified 4-miRNAs, including miR-191-5p, miR-214-3p, miR-451a, and miR-489, which were significantly associated with lymph node metastasis and patient survival in BC. A total of 40 BC samples and adjacent normal tissues were compared in terms of has-miR221-3p expression using quantitative real-time PCR [126]. Then, its expression was explored with clinicopathological factors. Higher expression levels of hsa-miR-221-3p were observed in BC tissues than in adjacent noncancerous breast biopsies (*p* ≤ 0.0001), but there was no significant correlation between hsa-miR-221-3p and the clinicopathological characteristics (*p* > 0.05). 

Wang et al. investigated the lncRNA signature for predicting recurrence among ER+ BC patients that were treated with tamoxifen using cohorts from Gene Expression Omnibus (GEO) (*n* = 298) and The Cancer Genome Atlas (TCGA) (*n* = 160), as training and validation cohort, respectively [127]. They represented 11 lncRNAs, including PINK1.AS, RP11.259N19.1, KLF3.AS1, LINC00339, LINC00472, RP11.351I21.11, KB.1460A1.5, PKD1P6.NPIPP1, PDCD4.AS1, KLF3.AS1 PP14571, and RP11.69E11.4 as reliable prognostic and predictive biomarkers for disease relapse in BC patients receiving tamoxifen.

In 2018, Zhang et al. analyzed the expression levels and clinical significance of miR-597 in 190 paired BC samples with noncancerous BC [128]. They showed low miR-597 expression in BC compared to adjacent non-tumor tissues (*p* < 0.001). A close correlation was found between low miR-597 expression with positive lymph node metastasis (*p* = 0.001), advanced TNM stage (*p* = 0.003), poorer tumor differentiation (*p* = 0.006), and unfavorable OS than cases with higher miR-597 expression levels (*p* = 0.009). It can be concluded that miR-597 can be an independent prognostic indicator of BC (*p* = 0.005; HR 2.273; CI 95%, 1.117–4.291). In a comprehensive study, 121 BC and 56 benign breast tissue specimens were compared in terms of miR-29b levels using quantitative real-time PCR [129]. Their findings revealed that MiR-29b expression did not show a significant difference between the two groups. However, decreased levels of MiR-29b were found in invasive ductal adenocarcinomas versus their lobular counterparts (*p* = 0.010). In addition, the overexpression of miR-29b was found in samples with ER+ (*p* = 0.021) in the overall population, whereas it was negatively correlated (*p* = 0.035) with primary tumor staging in the ductal subset and increased in poorly-differentiated tumors of lobular origin (*p* = 0.041). Of note, BC cases with ductal carcinoma and increased levels of miR-29b had a significantly longer disease-free survival (*p* = 0.010) and a lower risk to relapse (HR = 0.35, 95% CI, 0.15–0.81; *p* = 0.014).

In 2018, the expression levels of miR-301a were compared in 380 BC samples, including non-TNBC and TNBC specimens, using in situ hybridization (ISH) [130]. Then, they validated the role of miR-301a as an independent prognostic factor in BC cases using public breast cancer databases, such as TCGA and METABRIC. Their findings showed that higher expression of miR-301a in BC cases is correlated with reduction of five-year DFS and OS compared to BC with low levels of miR-301a expression. It can be assumed that miR-301a can provide novel therapeutic options in BC cases with overexpression of miR-301a to reduce recurrence and the mortality rate.

Zidan et al. evaluated 80 BC cases compared to 80 controls in terms of MALAT1 expression using RT-quantitative polymerase chain reaction (qPCR) and CA15-3 using chemiluminescence immunoassay (CLIA) [131]. Elevated expression of MALAT1 was significantly found in BC samples compared to controls (*p* < 0.0001). Diagnostic sensitivity and specificity for BC were 83.7% and 81.2%, respectively, for MALAT1 expression and 77.5% and 82.5%, respectively, for CA15-3 level. Interestingly, a positive correlation was observed between MALAT1 expression with lymph node status, ER status, tumor stage, and histological grade indicating its possible prognostic value. 

Liu et al. used Gene Chips analysis and found higher expression of lncRNA00544 in the metastatic axillary nodes compared to luminal BC tissues (fold change = 2.26, *p* = 0.043) [132]. They confirmed these results in luminal BC cell lines (*p* = 0.0113) and 49 paired BC samples versus controls (*p* = 0.011). Elevated expression of lncRNA00544 was correlated with poor disease-free survival. It can be reflected that lncRNA00544 can represent a novel predictive and prognostic biomarker in luminal BC patients.

Clinical significance of miR-9 and miR-155 was explored in 190 resected TNBC specimens using qRT-PCR [133]. Then, Wang et al. evaluated 669 patients without de novo stage IV TNBC the relationship between expression of these miRNA and EMT marker expression, including vimentin, smooth muscle actin [SMA], osteonectin, N-cadherin, E-cadherin, CD146, and ZEB1 examined by qRT-PCR and confirmed with immunohistochemistry. A positive correlation was seen between miR-9 with the pT category, whereas there was no significant association between miR-155 expression and clinicopathologic features of TNBC. No correlation was found between miR-9 expression with EMT marker expression except for SMA, whereas expression of miR-155 showed an inverse relationship with EMT markers. In conclusion, increased miR-9 levels showed significant association with poor PFS and distant metastasis–free survival (DMFS) in TNBC, whereas high level of miR-155 expression was associated with better DMFS. Their study suggests that the expression levels of both miR-9 and miR-155 can serve as candidates for prognostic biomarkers in the TNBCs that were evaluated [134]. Then, diagnostic and prognostic values of candidate miRNAs were confirmed in the training and validation cohorts, respectively. Additionally, their biological significance was assessed using the bioinformatic analysis, in vitro and in vivo assays. In the discovery set, they showed up-regulation of miR-629-3p in metastatic foci (fold change 144.16, *p* < 0.0001) and primary tumors of TNBC patients with lung metastases (fold change 74.37, *p* = 0.004). In the training set, the ROC curve indicated that miR-629-3p had high diagnostic accuracy in discriminating patients with lung metastasis from patients without recurrence (AUC 0.865, 95% CI 0.800–0.930, *p* < 0.0001). miR-629-3p was correlated with poor OS and DFS in the validation set, but it failed to show significance after multivariate analysis. Surprisingly, logistic regression analyses demonstrated that miR-629-3p was an independent risk factor for lung metastasis (OR 4.1, 95% CI 2.5–6.6, *p* < 0.001). Ablation of miR-629-3p showed decreased viability and migration of TNBC cells, and it markedly suppressed lung metastasis in vivo. The authors also reported the leukemia inhibitory factor receptor (*LIFR*), a well-known metastatic suppressive gene, to be a direct target of miR-629-3p.

The expression of miR-101 in 781 patients with BC from The Cancer Genome Atlas (TCGA) were examined [135]. Gene expression profiling of GSE31397 with miR-101-3p transfected MCF-7 cells and scramble control cells were downloaded from the Gene Expression Omnibus (GEO), and the differentially expressed genes (DEGs) were identified. Based on TCGA analysis from 781 BC patients, low levels of miR-101-2 expression might represent a diagnostic (AUC: 0.63) marker, whereas the miR-101-1 was a prognostic (HR = 1.79) marker. There was a close correlation between ER, PR, and HER2, while miR-101-2 was correlated with the tumor (T), lymph node (N), and metastasis (M) stages of BC. More than 400 genes were selected from the 921 DEGs in GEO and the 7924 potential target genes from the prediction databases. The authors showed that these genes were related to transcription, metabolism, biosynthesis, and proliferation. Their results were also significantly enriched in the VEGF, mTOR, focal adhesion, Wnt, and chemokine signaling pathways.

Jiang et al. used a high-density SNP array-based approach to uncover intergenic dysregulated lncRNA genes that were involved in BC [136]. Then, the role of LincIN in BCC progression and metastasis was evaluated using an in vitro invasion assay and a mouse tail vein injection metastasis model. The target genes of LincIN were identified by RNA pull-down experiments followed with protein identification by mass spectrometry. The overexpression of LincIN was more often seen in BC versus adjacent normal tissues and was closely associated with BC aggressiveness and shorter OS (*p* = 0.044 and *p* = 0.011 after adjustment for age). More interestingly, ablation of LincIN showed inhibition of tumor cell migration and invasion in vitro and diminished lung metastasis in a mouse tail vein injection model. The authors also showed that NF90, LincIN-binding protein, can suppress the expression of p21 protein by inhibition of its translation. 

Analysis of SPRY4-IT1 in 110 of BC and adjacent normal breast tissues were evaluated by quantitative real-time PCR (qRT-PCR) and then its association with clinicopathological parameters was analyzed [137]. Increased expression of Z38 was found in BC compared to controls, advanced TNM stage, presence of lymph node metastasis, and unfavorable OS. 

A total of 64 BC patients with large tumors or locally advanced with neoadjuvant anthracycline/taxane-based chemotherapy examined for 10 miRNAs, including (miR-7, -21, -29a, -29b, -34a, -125b, -155, -200c, -340, -451) that were likely to be associated with chemotherapy response [138]. Wu et al., showed that the patients with miR-7low or miR-340 high profile might not have complete response (pCR) in 19 BC patients. The expression patterns and clinical significance of miR-199b-5p in BC and non-cancerous breast tissues were detected by qRT-PCR [139]. The authors showed down-regulation of MiR-199b-5p in BC versus adjacent normal tissues (*p* < 0.05). Low expression of MiR-199b-5p showed close association with advanced TNM stage (*p* = 0.008), positive lymph node metastasis (*p* = 0.013) and poor OS (HR = 2.318, 95%CI = 1086–4949, *p* = 0.030). It can be assumed that miR-199b-5p might be a possible marker for BC. 

Huang et al. evaluated mRNA expression of MALAT1 in BC cell lines and 33 pairs of primary non-metastatic ER+ BC and their matched adjacent normal tissues samples [140]. Seshandri et al. analyzed the clinical significance of MALAT1 in a large sample of 1014 BC samples. The up-regulation of MALAT1 was found in all BC cell lines except MCF10A cells as well as ER+ BC samples versus adjacent normal tissues (*p* = 0.012). They also showed a close correlation between MALAT1 and positive status of ER (*p* = 0.023) and progesterone receptor (PR) (*p* = 0.024). Analysis of the TCGA database indicated that ER and its target genes *PGR* and *CCND1*, increased in the MALAT1-altered group compared to the unaltered group, both on the mRNA and protein level. Significantly, up-regulation of MALAT1 was associated with poor RFS in tamoxifen-treated ER-positive BC patients, which might present as a candidate biomarker to predict endocrine treatment sensitivity.

The significance of 12 circulating miRNAs in the serum of inflammatory and non-inflammatory BC was evaluated in a Tunisian population [134]. Meseure et al. analyzed MALAT1 expression patterns and its clinical significance in a large collection of BC specimens using RT–PCR, in situ hybridization, and RPPA methods The authors demonstrated that the overexpression of miR-335 in 446 unilateral premenopausal non-inflammatory BC patients, whereas miR-24 was significantly up-regulated in non-inflammatory BC with postmenopausal status. Higher miR-342-5p was found in BC cases with previous parity compared to without parity. Lower levels of miR-15a were seen in inflammatory BC samples with HER2+ versus HER2-. [142]. Their findings revealed the overexpression of MALAT1 in 14% of BC samples. In addition, a major alternatively spliced MALAT1 transcript, Δsv-MALAT1, was mainly under-expressed in nearly 19% of BC patients. Therefore, the authors reported a complex expression pattern of various MALAT1 transcript variants in BC cases and the prognostic and predictive role of MALAT1 should be considered conservatively. In 2016, the researchers assessed the local control in early stage BC after breast conserving therapy (BCT) [143]. At first, 32 patients (16 relapses versus 16 controls) were screened for the most dysregulated microRNAs in a panel of 1250 miRNAs using microarrays. Then, eight candidate miRNAs were tested in 115 patients (30 relapses versus 85 controls) with RT-qPCR. From these eight candidates, hsa-miR-375 could be validated. They demonstrated a positive association between the levels of hsa-miR-375 with local relapse (*p* = 0.003). Zehentmayr et al. also showed that hsa-miR-375 can distinguish between relapse and control groups (raw *p*-value = 0.000195 HR = 0.76, 95% CI 0.66–0.88; corrected *p*-value = 0.005).

In 2016, miR-520 expression and its clinical relevance were evaluated in the peripheral blood of 86 cases with breast cancer (including 18 cases with stage 0, 24 cases of Stage I, 20 cases of Stage II, 24 cases of Stage III) and 26 controls using real-time quantitative PCR (RT qPCR) [144]. Madhavan et al. undertook a comprehensive study made of 67 metastatic BC and 265 non metastatic BC patients through taqman low density array, 16 miRNAs, including miR-141, miR-144, miR-193b, miR-200a, miR-200b, miR-200c, miR-203, miR-210, miR-215, miR-365, miR-375, miR-429, miR-486-5p, miR-801, miR-1260, and miR-1274a, showed a significant correlation with OS in BC cases. Higher levels of miR-520g were found in BC patients with lymph node metastatic (*p* = 0.033) and low differentiation degree grade (*p* = 0.016), mammary gland invasion (*p* < 0.01), and low expression of p53 (*p* = 0.0039). It can be reflected that miR-520g might be a potential prognostic factor in BC. A significant correlation was found between miR-200a, miR-200b, miR-200c, miR-210, miR-215, and miR-486-5p with metastasis development before clinical manifestation of BC [145].

A total of 92 BC tissues and adjacent normal tissues were compared in terms of lncRNA CCAT1 using quantitative real-time PCR. A significantly higher expression of lncRNA CCAT1 in BC tissues was observed in BC tumors versus adjacent normal tissues. In addition, there was a significant correlation between CCAT1 with poor differentiation grade, advanced TNM stage, presence of lymph node metastases, and shorter OS and PFS indicating that lncRNA CCAT1 could be a possible prognostic marker for BC progression [146].

The expression patterns and clinical relevance of miR-124 were measured using quantitative real-time PCR in 133 BC patients. Lower expression levels of miR-124 was seen in the BC samples compared to adjacent normal breast tissues (0.39 ± 0.16 vs. 1.00 ± 0.39; *p* < 0.05). In addition, Dong et al. demonstrated a positive correlation between low expression of miR-124 with advanced TNM stage (*p* = 0.011), lymph node metastasis (*p* = 0.012), poorer pathological differentiation (*p* = 0.023), and shorter OS (63.8% vs. 35.2%, *p* = 0.03). Thus, this group showed that miR-124 can be an indicator of tumor progression and poor prognosis in BC cases [147]. In 2015, lncRNA microarray data from 164 primary breast tumors from adjuvant naïve patients were analyzed. A total of 82 patients’ cases with detectable distant metastasis were compared to 82 patients with no metastases. These results revealed that lncRNA profiles could distinguish metastatic patients from non-metastatic patients with sensitivities above 90% and specificities of 64–65% [148].

The prognostic value of HOTAIR was tested in 133 BC cases using RNA an in situ hybridization (RNA-ISH) assay. Then, these data were validated in a large collection of BC subjects that were obtained from The Cancer Genome Atlas (TCGA). The authors reported no correlation between the expression of HOTAIR with clinicopathological factors. In the TCGA dataset, HOTAIR expression was lower in ductal carcinomas but higher in the ER−BC samples. In this dataset, the overexpression of HOTAIR did not show correlation with nodal metastases or prognosis in ER + BC patients. Interestingly, their results indicated that HOTAIR might be an indicator of lymphatic metastases rather than hematogenous metastases in ER−BC [149]. 

Real-time quantitative (RQ)-PCR was applied to measure the miR-21 expression in serum, tumor tissue, and adjacent normal tissue from 549 cases (326 with breast cancer, 223 without breast cancer) [150]. Increased expression of microRNA-21 (miR-21) was reported in tissues and serum of BC patients versus healthy control groups in the Chinese population. Also, their findings indicated that serum miR-21 can be an indicator of recurrence (HR = 2.942; 95% CI = 1420–8325; *p* = 0.008) and disease-free survival (HR = 2732; 95% CI = 1038–7273, *p* = 0.003) in BC.

In a study that was conducted by Zheng et al., the expression levels of miR-106b and its clinical relevance was tested in both tissue and plasma samples of 173 patients with primary BC and a set of 50 women with fibroadenoma [151]. Increased levels of miR-106b were reported in both tissue and plasma BC samples as well as in larger tumor size, higher Ki67 expression, lymph node metastasis (all *p* < 0.05), and shorter PFS and OS (*p* < 0.001). It is clear that miR-106b might indicate a high risk of recurrence of BC. Quantitative in situ hybridization assay (qISH) was used to measure miR34a in three independent primary BC cohorts (Cohort 1 with 461, Cohort 2 with 279, and Cohort 3 with 795 patients) [152]. A close association between the loss of miR34a and poor outcome was observed in three independent breast cancer cohorts (uncorrected log-rank *p* = 0.0188 for Cohort 1, log-rank *p* = 0.0024 for Cohort 2, and log-rank *p* = 0.0455 for Cohort 3). In all the cohorts, a loss of miR34a can distinguish patients with poor PSF among node-negative patients, but not in the node-positive population. In conclusion, a loss of miR34a might be an indicator of a subgroup of BC patients with unfavorable disease-specific survival. 

Müller et al. investigated the relevance of miR-21, miR-210, and miR-373 in the serum of 127 HER2+ BC patients before and after chemotherapy combined with either trastuzumab or lapatinib treatment as well as compared to 19 healthy controls [153]. Higher levels of miR-21 (*p* = 5.04 × 10^−8^, *p* = 1.43 × 10^−10^), miR-210 (*p* = 0.00151, *p* = 1.6 × 10^−5^), and miR-373 (*p* = 7.87 × 10^−6^, *p* = 1.75 × 10^−7^) were significantly observed in the serum of patients before and after chemotherapy compared to healthy women. The correlation of miR-21 levels before (*p* = 0.0091) and after (*p* = 0.037) chemotherapy with the OS of the patients could be detected, independent of the type of anti-HER2 therapy. It can be concluded that there is a close association between neoadjuvant therapy with the serum levels of miR-21, miR-210, and miR-373 in BC cases with a prognostic value of miR-21.

A training set of 58 patients and a validation set of 41 patients with invasive ductal TNBC were assessed in terms of prognostic signature with qRT-PCr [154]. Only lymph node status showed a marginal trend with poor prognosis of TNBC (*p* = 0.054). Expressions of miR-27b-3p, miR-107, and miR-103a-3p were significantly up-regulated in the metastatic group versus the disease-free group (*p* = 0.008, 0.005, and 0.050, respectively). In addition, the authors demonstrated that lymph node status and miR-27b-3p were independent predictors of poor prognosis (*p* = 0.012 and 0.027, respectively). A total of two different risk groups were stratified according to the model, showing significant differences in terms of distant metastasis and BC-related death in the training set (*p* = 0.001 and 0.040, respectively) as well as in the validation set (*p*: 0.013 and 0.012, respectively). 

The expression of miR-10b in BC tumor and paired normal specimens with at least 36 months follow-up was analyzed [155]. The relative expression of miR-10b in tumor versus its normal counterpart (RER) was analyzed by RT-qPCR. miR-10b RERs were higher in the patients with metastases (*n* = 11, median 0.25; IQR 0.11–1.02) compared to patients without metastases (*n* = 90, median 0.09; IQR 0.04–0.29) (*p* = 0.028). In patients without metastases (*n* = 90), overexpression of miR-10b RERs were associated with increased risk of disease progression and death in both univariable (HR 1.16, *p* = 0.021 and HR 1.20, *p* = 0.015, respectively, for 0.10 unitary increase of miR-10b RERs levels) and multivariable (HR1.30, *p* < 0.001, and HR 1.31, *p* = 0.003, respectively, for 0.10 unitary increase of miR-10b RERs levels). These data revealed that adding miR-10b RERs to the prognostic factors that are used in clinical routines could improve the prediction abilities for the overall mortality as well as progression in BC patients.

Corcoran et al. examined the levels of intra- and extracellular miR-630 in cells and conditioned media from BC cell lines and 56 BC tissues with either innate- or acquired- resistance to HER-targeting lapatinib and neratinib, versus corresponding drug sensitive cell lines, using qPCR [156]. Additionally, the clinical relevance of miR-630 was explored in BC tumors compared to matched peritumor specimens. To evaluate the effects of miR-630 on the response to HER-targeting drugs (lapatinib, neratinib, and afatinib), the BC cells were transfected with miR-630 mimics and inhibitors. The induction of miR-630 into cells with innate- or acquired-resistance to HER-drugs significantly restored the efficacy of lapatinib, neratinib, and afatinib with mediated of IGF1R, whereas the inhibition of miR-630 induced insensitivity to these agents. This study clarified the role of miR-630 as a diagnostic and a predictive indicator for response to HER-targeted drugs. 

Pérez-Rivas et al. tested 71 primary BC samples that either remained disease-free at five years post-surgery (group A) or developed early (group B) or late (group C) recurrence using microarray-based technology and qRT-PCR [157]. A total of five microRNAs, including miR-149, miR-10a, miR-20b, miR-30a-3p, and miR-342-5p, decreased in patients with early recurrence. In addition, these five 5-miRNA signatures determined a high-risk group of patients with shorter relapse-free survival as well as non-relapsing versus early-relapsing patients (AUC = 0.993, *p* < 0.05). It can be reflected that these recurrence-related microRNAs have a possible prognostic value to identify patients with metastasis development after primary breast surgery.

In 2014, Gasparini et al. evaluated the miRNA expression profile and found a subset of miRNAs that were specifically deregulated in the two subclasses, within 160 TNBC first with GEO and TCGA and then validated with qRT-PCR [158]. They identified 4-miRNA, including miR-155, miR-493, miR-30e, and miR-27a that allowed subdivision of TNBCs not only into CB and 5NP subgroups (sensitivity 0.75 and specificity 0.56; AUC = 0.74) but also into high risk and low risk groups. Then, they tested the diagnostic and prognostic value of both the five IHC marker panel (CB, EGFR and/or CK5, 6 positive) and the 4-miRNA expression signatures, which distinguish worse outcome patients in the treated and untreated groups. It is clear that TNBC subclassification based on the five IHC markers and on the miR-155, miR-493, miR-30e, and miR-27a expression levels are a powerful diagnostic approach.

The expression of Ataxia telangiectasia-mutated (ATM) and miRNAs, including miR-26a, miR-26b, miR-203, miR-421, miR-664, miR-576-5p, and miR-18a, was analyzed by RT-qPCR in 52 BC and three normal breast samples [159]. Protein expression of ATM was assessed by immunohistochemistry in 968 BC and 35 adjacent normal breast tissues. In addition, ATM copy number alteration was determined by array comparative genomic hybridization (aCGH) in 42 tumors. A lack of ATM protein expression was correlated with distant metastasis (*p* < 0.001), reduced disease-free survival (DFS, *p* < 0.001), and cancer-specific survival (CSS, *p* < 0.001). The authors showed that ATM protein expression was an independent prognostic marker for DFS (*p* = 0.001, HR = 0.579) and CSS (*p* = 0.001, HR = 0.554). A loss of ATM copy number was observed in 12% of tumors and was associated with lower mRNA levels. The increased expression of miR-421 was detected in 36.5% of cases which exhibited lower ATM transcript levels (*p* = 0.075, r = −0.249). It is clear that ATM protein expression might represent an independent prognostic marker in sporadic BC. 

The researchers examined the expression levels of HOTAIR using a microarray in 164 primary BC without adjuvant therapy [160]. Their findings revealed the differences in HOTAIR expression between patients with or without a metastatic endpoint, respectively. A significant correlation was reported between high HOTAIR expression with worse prognosis (*p* = 0.012, HR = 1747). A stronger association was seen in cases with ER+ (*p* = 0.0086, HR 1985), but not in ER− tumor samples. Overall, it can be concluded that HOTAIR expression may provide an independent biomarker for the prediction of the risk of metastasis in ER+ BC patients. 

In a prior study, the expression of CCAT2 was explored in BC and normal breast tissues using RT-qPCR and ISH [161]. Then, the authors explored the clinical relevance of CCAT2 expression in an independent set of 997 primary BC. They demonstrated the highest expression of CCAT2 in patients with an absence of lymph node involvement. Additionally, it has been shown that CCAT2 upregulates cell migration and down-regulates chemosensitivity to 5’FU in a rs6983267-independent manner. 

In a previous study, the expression patterns of miR-27a and ZBTB10 were analyzed in 102 BC cases using in situ hybridization (ISH) and immunohistochemistry techniques [158]. Then, the clinical relevance of these markers was explored with clinicopathological factors. The authors showed a reverse correlation between miR-27a and ZBTB10 in BC tissue samples (r(s) = −0.478, *p* < 0.001). Patients with high miR-27a or low ZBTB10 expression showed significantly shorter DFS (57 months and 53 months, respectively, *p* < 0.001) and OS (58 months and 55 months, respectively, *p* < 0.001). Univariate and multivariate analysis showed that both miR-27a and ZBTB10 were independent prognostic factors of disease-free survival in breast cancer patients (*p* < 0.001), while only miR-27a was an independent predictor of the overall survival (*p* < 0.001). It is clear that miR-27a could be considered as a valuable marker of BC progression.

Yan et al. immunostained 94 familial breast cancers (28 BRCA1, 27 BRCA2, and 39 BRCAX) to evaluate RAD21 expression [162]. There were no significant differences in the nuclear RAD21 expression between BRCA1 (12 (43%) of 28), BRCA2 (12 (44%) of 27), and BRCAX cancers (12 (33%) of 39 (*p* = 0.598). The authors showed no correlation between RAD21 expression with grade, size, or lymph node, ER, or HER2 status (all *p* > 0.05). In addition, RAD21 expression was correlated with shorter survival in Grade 3 (*p* = 0.009) and but not in Grade 1 (*p* = 0.065) or 2 cancers (*p* = 0.090). Shorter survival was reported between RAD21 expression and cases who received chemotherapy (*p* = 0.036) but not with hormonal therapy (*p* = 0.881). Interestingly, RAD21 expression correlated with shorter survival in BRCA2 (*p* = 0.006) and BRCAX (*p* = 0.008), but not BRCA1 cancers (*p* = 0.713). Changes in RAD21 mRNA were evaluated by genomic changes in DNA copy number (*p* < 0.001), whereas RAD21 protein expression was analyzed with immunohistochemistry (*p* = 0.047). Increased RAD21 expression was correlated with genomic instability based on the total number of base pairs that were affected by genomic change (*p* = 0.048). Of the 15 miRNAs that were predicted to target RAD21, mir-299-5p inversely correlated with RAD21 expression (*p* = 0.002). In conclusion, RAD21 is a potential predictive and prognostic biomarker in familial breast cancers. 

The expression of let-7b and miR-205, two most frequently lost miRNAs in a wide range of malignant tumors, was evaluated in 2919 BC using tissue microarrays (TMAs) [163]. Based on the ER, PR, HER2, CK5/6, and EGFR expressions, BC was divided into different subtypes. The authors showed that the expression of miR-205 is associated with tumors of ductal morphology and thus this molecule can be considered as a prognostic marker within these tumors. 

Rothé et al. analyzed miRNA expression profiling of 56 systemically untreated BC patients firstly using microarray and secondly with qRT-PCR [164]. Then, these data were validated in an independent dataset of 89 ER+ BC patients who received only tamoxifen. The authors showed that miR-210 expression was associated with poor clinical outcome in ER+ as well as tamoxifen-treated BC patients. The effects of MiR-210 were analyzed on the BC cells, including MCF7 and MDA-MB-231. MiR-210 expression showed that this molecule was involved in cell proliferation, migration, and invasion.

In a preliminary study, microRNA pattern and its clinical significance were investigated in 103 lymph node-negative breast cancers. Based on an unsupervised hierarchy, the patients were divided into four main groups; the basal-like/triple-negative group was the most prominent (11% of all cases), the luminal A cancers containing the Her2- and ER+/PR+ tumors was the largest group (57%), and the group of luminal B (32%) was more heterogeneous and contained the Her2+/ER− patients as well. The MiR-106b gene was prominent in all these groups and showed a close correlation with high proliferation. The authors also demonstrated the presence of several microRNAs, including miR532-5p, miR-500, miR362-5p, and miR502-3p, located at Xp11.23 in cancers with a triple-negative signature, and the increased expression of several miR-17 cluster members in ER− tumors [165].

In a prior study, the researchers investigated the Dicer expression in 104 BC cell lines and tissue samples that were obtained from patients with long-term follow-up using TMAs and qRT-PCR [166]. Lower Dicer expression was found in the BC cell lines with a mesenchymal phenotype and in metastatic bone derivatives of a BC cell line. A close correlation was reported between Dicer protein expression and hormone receptor status and subtypes in BC (ER *p* = 0.008; PR *p* = 0.019; cancer subtype *p* = 0.023, luminal A *p* = 0.0174). It can be concluded that Dicer expression might be an indicator of distant metastases in BC cases. 

Tumor-specific mRNAs and lncRNAs were analyzed with a microarray in a training cohort that was made of a total of 198 frozen tissues from 165 consecutive TNBC patients (including 33 pairs of tumor and adjacent normal tissues) and a validation cohort made of 266 frozen TNBC samples and 33 adjacent normal breast tissue [167]. Tumor-specific mRNAs and lncRNAs were identified and correlated with patients’ recurrence-free survival (RFS). An mRNA and an mRNA-lncRNA signature based on eight mRNAs and two lncRNAs were established. In the training set, recurrence was more frequently found in the high-risk group compared to the low-risk group in both signatures (HR, 10.00; 95% CI, 2.53-39.47, *p* = 0.001; HR = 4.46, 95% CI, 1.34–14.91, *p* = 0.015 for integrated signature and mRNA signature, respectively). 

In a comprehensive study, the clinical relevance of *EPB41L4A-AS2* was evaluated in 250 BC tissues and 50 healthy tissues with qRT-PCR in mediating cancer cell proliferation in BC cell lines that were transfected with an *EPB41L4A-AS2* expression vector [168]. The authors found that high *EPB41L4A-AS2* expression was correlated with favorable disease outcomes. In addition, induction of *EPB41L4A-AS2* expression inhibited breast tumor cell proliferation. It can be concluded that evaluation of this long non-coding RNA might provide a possible prognostic biomarker in the clinical management of BC. 

Lánczky et al. developed a database called miRpower, by searching the GEO, EGA, TCGA, and PubMed repositories to detect datasets with previously published miRNA expression and clinicopathological data (using GEO, EGA, and TCGA) in 2178 BC patients [169]. Kaplan–Meier survival analysis was also used to validate the prognostic value of a set of 41 these miRNAs. The authors demonstrated that miR-29c and miR-101 might have prognostic value in BC patients. 

Hu et al. evaluated the role of miR-205 in prediction of TAC (docetaxol, doxorubicin, and cyclophosphamide) regimen in 30 BC patients [170]. The authors showed that the down-regulation of miR-205 in drug-resistant derivatives of MCF-7 and Cal51 cell lines and its induction sensitizes both drug-resistant cells to doxorubicin and taxol. In addition, they showed miR-205 inhibited vascular endothelial growth factor A (VEGFA) and fibroblast growth factor-2 (FGF2), resulting in decreased phosphatidylinositol 3-kinase (PI3K)/Akt signaling pathway activity and increased apoptosis upon chemotherapy. Thus, miR-205 may be valuable for the prediction of the TAC regimen as well as a possible therapeutic target in BC treatment.

The prognostic value of miRNA has been investigated in BC by Gong et al. The training group had 202 patients and two external validation cohorts of 308 samples with qRT-PCR. The following 10-miRNAs were a good prognostic biomarker to predict the distant relapse-free survival (DRFS) in BC: miR-7, miR-22, miR-21, miR-30c, miR-181a, miR-181c, miR-125b, miR-200a, miR-135b, and miR-200c. Overall, the signature outperformed traditional clinicopathological risk factors, 21-gene recurrence score (RS), and IHC4 scoring [171]. The prognostic value of non-coding RNA biomarkers for the prediction of tumor recurrence in BC patients has been investigated by Zhou et al. in 473 BC patients using GEO data. The authors identified 12 differentially expressed lncRNAs that were closely associated with tumor recurrence of BC from discovery cohort, which was able to classify patients into high-risk and low-risk with recurrence-free survival that was significantly different (HR= 2.72, 95% confidence interval 2.07–3.57; *p* = 4.8 × 10^−13^). In two out of three independent validation cohorts, the signature represented a similar prognostic value. Overall, the authors suggested that the signature deserves further attention and should be tested in more clinical settings [172]. The prognostic utility of lncRNA miRNA has been investigated together with that DNA methylation and mRNAs across five human cancers, including breast cancer, in 3198 samples from TCGA using an approach to prioritize ncRNAs called IDFO approach. Stunningly, lncRNA was the best prognostic predictor in the validated cohorts of four cancer types (including breast cancer), followed by methylation, mRNA, and then microRNAs [173]. The prognostic utility of an ER-associated miRNA signature has been investigated by Zhou et al. in ER+ BC. A total of two cohorts from TCGA dataset were used as training (*n* = 596) and testing set (*n* = 319). The authors saw that 14 miRNAs could be associated with the ER status by significance analysis of microarrays (SAM) in a training set. Patients could be characterized as high and low score according to the risk scores that were calculated for each miRNA. Patients with high score group had worse OS compared to patients with a low score in both the training and testing sets. Therefore, the signature could be used as prognostic marker in ER+ BC [174]. Dedes et al. investigated the prognostic value of miRNA master regulators Drosha and Dicer with breast cancer. The authors used qRT-PCR to screen 245 patients that were receiving adjuvant anthracycline-based chemotherapy to compare the expression levels to normal breast tissue. In 18% of cases Drosha down-regulation was associated with high grade, lack of Bcl2 expression, high Ki-67, HER2 over-expression and gene amplification, and TOPO2A gene amplification. The dicer down-regulation was found in 46% of cases and was associated with a lack of expression of PR, ER, and Bcl2 and high grade, high Ki-67, TNBC, and basal-like phenotypes. The authors observed a concurrent down-regulation of Drosha and Dicer in 15% of cases and a significant association with both high grade and ki-67 index. However, there was not a significant association between the down-regulation of Drosha and/or Dicer and outcomes [175].

## 4. Discussions

In recent years there has been an exhaustive and paramount amount of data from ncRNAs involvement in cancer biomarkers and therapy. Here we have analyzed the diagnostic and prognostic value of ncRNA for breast cancer. There is a plethora of miRNAs, lncRNA, Dicer ncRNA, circRNAs, and piRNAs that are summarized in Table 1 that have strong indications to be good prognostic biomarkers for predicting OS, tumor-size, TNM staging, lymph node metastasis, distant metastasis, and invasion depth. In our opinion, the capacity of the ncRNAs to act either as a positive or negative biomarker in BC depends on which specific pathways that have been targeted for inhibition or activation.

## 5. Conclusions

This comprehensive review shows that non-coding RNA could be used as diagnostic and prognostic biomarkers for breast cancer. Testing the capacity of these biomarkers in large and randomized clinical trials for immunotherapies and targeted therapies is another aspect that deserves to be further explored.

## Figures and Tables

**Table 1:** Non-coding RNA biomarkers for prediction of breast cancer prognosis. The clinical studies were searched on PubMed using the following words “biomarker non-coding RNA prognostic and diagnostic value in breast cancer”. We excluded animal studies, other reviews, and letters to the editor.

Reference Number	Non-Coding RNA/s Investigated	N° of Patients vs. Healthy Controls	Technique (TCGA, qRT-PCR or ddPCR)	Main Observation
[56]	6 lncRNAs (AC046168.1, AC010595.1, AC069277.1, AP000904.1 MIR762MG and LINC00528)	These RNA data were derived from 113 HER2-positive breast cancer tissues and 105 tumor-adjacent normal breast tissues.	TCGA	The 6-lncRNA model had a good predictive power for OS and 3-year survival in HER2+ BCs
[57]	miR-92b	112 female BC patients and 108 healthy women.	qRT-PCR	Higher expression level of miR-92b-3p. AUC of 0.88 correlated with degree of differentiation, tumor size and TNM staging, lymph metastasis. miR-92b significantly positively correlated with the expression of carbohydrate antigen 125 (CA125)
[58]	RP11-1024P17.1, RP11-890B15.3, MFI2-AS1 and RP11-180N14.1	Patients from TCGA and GEO databases.	TCGA and GEO databases	Useful to stratify patients into high and low risk groups and as prognostic biomarkers.RP11-890B15.3, RP11-180N14.1 and RP11-1024P17.1 could regulate more mRNAs by targeting various miRNAs. The MF12-AS1 regulated three mRNAs by sponging miR-3150a-3p.
[59]	lncRNA MIAT	1057 BC and 103 normal specimens	First: TCGA Then: qRT-PCR	Its expression in serum positively correlated with TNM stage and lymph node metastasis
[60]	miR-222	110 patients	qRT-PCR	Capacity to inhibit tumor suppressor CDK inhibitor p27 in BC. miR-222 was expressed at significantly higher levels in BC. Serum p27 and miR-222 could help differentiate between BC and controls
[61]	8-IncRNA	808 BC patients	First: TCGA Then: qRT-PCR	Potential prognostic biomarker for BC
[62]	LINC01871, MAPT-AS1, AL122010.1, AC090912.1, AC061992.1	1108 BC patients	TCGA	Their model offered an independent prognostic value, with a risk score significantly related to the TNM stage, PR, ER and HER2 status in BC patients
[63]	LINC01977, AP000851.1, MAFG-D, SIAH2-AS1	1222 BC patients	TCGA	Synergistically exerted functions related to cell cycle and DNA separation, DNA replication, and an independent prognostic marker for BC
[64]	IncRNA CARMN	250 BC patients	qRT-PCR	CAMN could predict both better prognosis and higher response rate of cisplatin-based neoadjuvant chemotherapy in BC patients and inhibit DNA replication.
[65]	miR-25-3p	25 BC patients	qRT-PCR	Patients with low expression levels of serum miR-25-3p had a higher survival compared to those with higher miR-25-3p expression. miR-25-3p could be a good biomarker for BC
[66]	PIWI-interacting RNAs (piRNAs): DQ570994, DQ571955, and DQ596932	227 fresh-frozen BC samples.	RNA sequencing	piRNSAs were upregulated in grade III tumors and DQ696932 was upregulated in estrogen receptor negative tumors only. DQ571955 showed shorter relapse-free survival and poorer BC-specific survival. DQ571955 can be a predictive biomarker for radiotherapy response in ER+ BCs. DQ570994 can be a predictive marker for tamoxifen and chemotherapy response
[67]	NEAT1 and MAL2	63 patients SCLC	GEO2R tool	Able to differentiate good prognosis vs. bad regression free survivals
[68]	ZFAS1	40 TNBC patients compared to 40 healthy individuals	qRT-PCR	ZFAS1 promoted proliferation of human breast cancer cell line MDA-MB-231 TNBC cells through the inhibition of the cyclin-dependent kinase (CDK) inhibitors p21 and p27. ZFAS1 could be a diagnostic and prognostic marker for TNBC that could be also used for therapy.
[69]	miR-182 and miR-18	50 TNBC patients	qRT-PCR	A significant correlation was observed with clinical nodal status, T-category, clinical response, pathological response with miR-18 and miR-182
[70]	miR153 miR-196a	Meta-analysis collecting data from 933 patients from 11 articles.	Meta-analysis	Low miR-153 expression significantly correlated with poor OS miR-153 could be a very effective biomarker for tumor prognosis especially in BC and digestive tumors.
[71]	miR-196a	17 articles were included	Meta-analysis	Tumor tissue or blood-derived miR-196a could be used a prognostic and diagnostic biomarker for cancers such as BC
[72]	miR-206	2095 patients	qRT-PCR	Potential prognostic biomarker. The pooled HR showed that low miR-206 expression was significantly associated with unfavorable OS Moreover, the expression of miR-206 predicted significantly negative association with tumor stage distant metastasis, lymph node status and invasion depth
[73]	miR-1225	120 BC patients	qRT-PCR	Significantly upregulated in BC and associated with TNM stage of BC. Overexpression of miR-1225 could be used as a biomarkers since it correlated with a poor prognosis of patients and promoted the progression of BC by targeting JAK1
[74]	TINCR	72 TNBC patients, 105 non-TNBC patients, 60 benign BC, and 86 heathy subjects	qRT-PCR	The lncRNA TINCR level was significantly increased in BC patients, particularly in TNBC. The clinicopathological features and clinical outcomes of TNBC were worse in patients with high circulating lncRNA TINCR
[75]	ADAMTS9-AS1, CDKN2B-AS1, IL-6, MMP11, has-miR-145-5p ahashsa-miR-182-5p	787 early BC patients and 78 normal BC individuals.	qRT-PCR	AUC were 0.947, 0.862, 0.842, 0.993, 0.960, and 0.944, and the specificity and sensitivity 83.4% and 95.6%, 72.2% and 90.3%, 80.1%, and 74.3%, 96.2% and 96.5%, 90.1%, and 92.3%, and 88.7%, and 90.4%, respectively
[76]	TPT-AS1	316 BC patients	First: TCGA and GEOSecond: qRT-PCR	The low expression of TPT1-AS1 was correlated with lymph node metastasis, TNM stage, HER-2- status, shorter OS. TPT1-AS1 was an independent prognostic factor for BC patients
[77]	miR-1246 and miR-155	107 early-stage and 68 metastatic BC patients treated with trastuzumab-based chemotherapy	Meta-analysis	Both miRNAs were not associated with OS. The study showed that miR-146 and miR-155 could distinguish trastuzumab-resistant from sensitive patients
[78]	9-IncRNA	10,213 BC patients	Meta-analysis	The 9-lncRNA signature was a robust and effective model for the prediction of DRFS of patients with HER2- BC
[79]	miR-20b	123 BC patients	qRT-PCR	Expression of miR-20b increased with increase in tumor grade and correlated with stromal overgrowth, high stromal atypia and cellularity, infiltrative tumor margin, tumor grade, high mitotic activity, local recurrence and metastasis, and shorter DFS miR-20b could be used as a prognostic biomarker in BC
[80]	miR-148a	125 patients with BC and 50 patients with benign breast tumors	qRT-PCR	miR-148a is significantly reduced in BC patients and down-regulation of serum exosomal miR-148a was closely associated with unfavorable clinical outcome of BC.
[81]	miR-1204	144 BC patients and 38 healthy volunteers	qRT-PCR	The study suggested that miR-1204 could be used a prognostic and diagnostic biomarker for BC7
[82]	SNHG6	914 patients from 13 studies	Meta-analysis	Expression could predict unfavorable OS. elevated level of SNHG6 was positively associated with tumor invasion depth, DM LNM and advanced TNM stage
[83]	miR-21	10,213 cancer patients	Meta-analysis	miR-21 correlated with shorter OS in breast cancer patients and could be a clinically useful biomarker for cancer progression.
[84]	Seven core lncRNAs. LINC00478, AL035610.1, AC005550.4, MIR143HG, MIR497HG, PGM5-AS1, RP11-175K6.1	837 BC	TCGA	Good single-factor diagnostic value for BC. Moreover, AC005550.2 had a prognostic value for BC. AC005550.4 and MIR497HG were able to distinguish better BC patients in early stage from patients at advanced stages. Overall, the 7-lncRNA could have a prognostic value in BC
[85]	Has-mir-124	742 BC patients	TCGA	OS of patients with high expression levels of has-mir-124-1 and has-mir-124-2 was better than that of patients with low expression levels of has-mir-124-1 and has-mir-124-2. Overall, has-mir-124 was associated with worse clinical outcomes and could be used as a biomarker for BRCA
[86]	miR-1179	164 BC patients	qRT-PCR	miR-1179 was frequently down-regulated in BC tissues and cell lines. low miR-1179 levels correlated with advanced clinical stage, shorter OS and lymph node metastasis Overall, miR-1179 could serve as a new prognostic biomarker or actionable target for new therapies
[87]	lncRNA in BC antiestrogen resistance 4 (BCAR4)	1293 patients from 9 studies	Meta-analysis	BCAR4 expression was significantly correlated with poor, and high expression levels of BCAR4 correlated with worse clinical stage, distant metastases, lymph node metastasis.
[88]	miR-93	Meta-analysis	qRT-PCR	The AUC for overall sensitivity and specificity were 0.76, 0.82, and 0.85), suggesting that miR-93 is a good prognostic biomarker.
[89]	3-miRNA signature (miR-133a-2, miR-204, and miR-301b).	1103 BC patients vs. 104 healthy samples	TCGA	The 3-miRNA signature could be a potential biomarker for BRCA
[90]	miR-2031-3p	1077 BC tissues compared to 104 adjacent BC samples	TCGA	miR-2031-3p did not have prognostic value in BC. Overall, the study suggests that miR-2031-3p could enhance tumorigenesis in BC, but has not prognostic utility.
[91]	Three miRNAs risk score (miR-19a, miR-93, and miR-106a)	1051 BC patients	TCGA	The miRNA-based risk score could predict worse survival and bone recurrence in BC patients
[92]	lncRNA maternally expressed gene 3 (MEG3)			The MEG3 was down-regulated in BC than in normal tissues. ER and progesterone receptor (PR) status positively correlated with MEG3 expression. MEG3 positively correlated with heparin sulfate proteoglycan 2 (HSPG2) expression and could be a good predictor of prognosis in BC with HSPG2.
[93]	2150 DEmRNAs, 1061 DElncRNAs, and 82 DEmiRNAs	1103 BC vs. 104 adjacent normal breast tissues	TCGA	The 4-lncRNA signature could independently predict OS in BC patients. The AUC of the 4-lncRNA signature associated with 3-year of survival was 0.696. Overall, the 4-lncRNA is a good prognostic tool for BC.
[94]	AC091043.1, AP000924.1, and FOXCUT.AC010343.3, AL354793.1, and FGF10-AS1	1097 BC samples	TCGA	AC091043.1, AP000924.1, and FOXCUT, may have a strong diagnostic value for the prediction of TNBC in both training and validation sets (AUC > 0.85).The signature could be efficient in predicting diagnosis and prognosis of TNBC
[95]	TUBA4B	94 BC patients vs. 86 normal tissues	Meta-analysis	TUBA4B is significantly correlated with OS DFS and recurrence-free survival. Overall, the study suggested that low levels of TUBA4B are significantly associated with short OS, DFS, and RFS in cancers and that TUBA4B could therefore be a BC biomarker
[96]	hsa-let-7b, hsa-mir-9-3, hsa-mir-22, hsa-mir-30a, hsa-mir-31, hsa-mir-101-2, hsa-mir- 135a-2, hsa-mir-320b-1, hsa-mir-493, hsa-mir-556, hsa-mir-652, hsa-mir-874, hsa-mir-3130-1, hsa-mir-3678, hsa-mir-4662a, hsa-mir-4772 and hsa-mir-6733. On the other hand sa-mir-130a, hsa-mir-204, hsa-mir-217, hsa-mir-223, hsa-mir-24-2, hsa-mir-29b- 1, hsa-mir-363, hsa-mir-5001, hsa-mir-514a-1, hsa-mir-624, hsa-mir-639, hsa-mir-659, and hsa-mir-6892	1098 BC	TCGA	The ROC analysis validated the accuracy and stability of these two signatures as an independent prognostic indicators for BC patients.
[97]	circRNA OMA1	64 pairs of BC tissues and adjacent normal tissues	qRT-PCR	Associated with tumor size and lymph node metastasis. circOMA1 promoted viability, migration, and invasion of BC cells. circOMA1 promoted tumor progression by upregulating sirtuin 4 (SIR4) and miR-1276. circOMA1 together with mir-1276/SirT4 could be prognostic markers for BC
[98]	miR-10b	61 BC patients and 48 healthy volunteers	qRT-PCR	The expression levels of serum miR-10b was progressively up-regulated in advanced stages of BC with higher levels in metastatic BC. Overall, the study evinced that simultaneous detections of miRNA10b and E-cadherin could be a robust serum biomarker to determine diagnosis and prognosticate BC metastasis.
[99]	SPRY4IT1	93 BC patients	qRT-PCR	SPRY4-IT1 was significantly expressed at higher levels in cancer tissues than normal tissues and its higher expression correlated also with higher rates of lymph node metastasis and recurrence and poor clinical response SPRY4-IT1 expression was a prognostic biomarker of poor clinical response in BC.
[100]	miR-923 and CA 15-3	253 BC patients	ddPCR	The miR-923 and CA 15-3 were significantly associated with prognosis. Combination of miR-923 and CA 15-3 detected from serum are a good preoperative, non-invasive prognostic marker of BC.
[101]	miR-138	30 BC and 10 healthy individuals	ddPCR	The level of miR-138 increased significantly in TNBC and correlated with poor prognosis. miR-138 was a good diagnostic and prognostic biomarker of TNBC.
[102]	ncRNA plasmacytoma variant translocation 1 (PVT1)	3,974 patients (39 articles).	Meta-analysis	PVT1 correlated with low OS TNM stage, tumor size, lymph node metastasis, and distant metastasis.
[103]	HOTAIR	15 BC patients treated surgically and 15 healthy individuals enrolled as controls; 25 BC patients received neoadjuvant chemotherapy before surgery and another 25 BC patients received tamoxifen hormone treatment after surgery	qRT-PCR	Potential diagnostic and prognostic biomarker since its high expression correlated with poor response to neoadjuvant chemotherapy and tamoxifen response
[104]	miR-155, miR-320a, and miR-205	28 studies	qRT-PCR	miR-155, miR-320a, and miR-205 could provide just some information on the major clinical-pathological features of BC, but not being used as a biomarker
[105]	miR-34	114 TNBC and blood samples from 124 healthy donors	IHC	TC and CC alleles associated with unfavorable prognosis in TNBC and that they could be used as a prognostic biomarker
[106]	miRNA-mediated prognostic modules		ProModule	New computational approach
[107]	hsa-miR-10a, hsa-miR-18a, hsa-miR-135b and hsa-miR-577	1098 TNBC	TCGA	The 4-miRNA signature was an independent prognostic factor of clinical variables in TNBC patients andit could be used as a prognostic biomarker for TNBC patients
[108]	ACTA2-AS1, RP11-384P7.7, RP11- 327J17.9, RP11-124N14.3, and RP11-645C24.5	511 breast cancer tissues vs. 59 normal tissues	TCGA	Potential biomarkers to predict potential diagnosis and prognosis of BC
[109]	circmiR-122	90 BC and 60 healthy controls	qRT-PCR	miR-122 could predict metastasis at a cutoff value of 10.9 with a sensitivity of 95.83% and a specificity of 65.15%. miR-122 expression could be a biomarker for OS and PFS
[110]	miR-16, miR-145, miR-155, miR- 451a, miR-21 and miR-486	38 luminal A BC patients vs. 20 healthy controls	qRT-PCR	The absolute value of the three miRNAs had a prognostic value in luminal A BC
[111]	IncRNA BCAR4	890 BC	Meta-analysis	High lncRNA BCAR4 expression correlated with poor OS. The higher levels of lncRNA BCAR4 significantly correlated with increased tumor stage, lymph node, and distant metastases
[112]	miR-331	130 malignant and 66 benign breast cancer surgically resected from primary tumors	q-RT-PCR	miR-331 significantly correlated with malignant breast tumors compared to their benign counterparts. miR-331 could be considered a good prognostic marker for BC.
[113]	miR-134, miR-125b-5P, miRNA-30a, miR-10a-5p and miR-222	176 BC patients	qRT-PCR	These 5 miRNAs could be used to predict distant recurrence during tamoxifen treatment.
[114]	LINC01296	55 BC patients	RT-qPCR	LINC01296 could be a negative prognostic biomarker that could be used to predict disease progression as well as an actionable target.
[115]	miR-940	128 BC patients	qRT-PCR	miR-940 could be an independent prognostic factor and a reliable biomarker for diagnosis and prognosis of BC.
[116]	HOTAIR	112 BC patients	qRT-PCR	The high expression levels of HOTAIR correlated with response to neoadjuvant chemotherapy as well as to a worse BC prognosis.
[91]	miR-330-3p	233 BC patients	qRT-PCR	miR-330-3p upregulation associated with prognosis in BC patients, suggesting that it could be a prognostic biomarker and an actionable treatment.
[92]	ncRNA variants associated with BC profiles	930 BC patients	TCGA	The authors observed that the overall mutation rate in coding and non-coding regions were significantly higher in ER− /HER2+ tumors
[93]	miR-191-5p, miR-214-3p, miR-451a, and miR-489	449 BC patients	qRT-PCR	Higher expression levels of hsa-miR-221-3p was observed in BC tissues than in adjacent healthy tissuebut there was no significant correlation between hsa-miR-221-3p and clinicopathological characteristics
[94]	hsa-miR221-3p	40	qRT-PCR	Higher expression levels of hsa-miR-221-3p was observed in BC tissues than in adjacent non-cancerous breast biopsies
[95]	PINK1.AS, RP11.259N19.1, KLF3.AS1, LINC00339, LINC00472, RP11.351I21.11, KB.1460A1.5, PKD1P6.NPIPP1, PDCD4.AS1, KLF3.AS1 PP14571, RP11.69E11.4	298 from GEO and 160 from TCGA	TCGA	Reliable prognostic and predictive biomarkers for disease relapse in BC patients receiving tamoxifen.
[96]	miR-597	190 paired fresh BC and non-cancerous BC	qRT-PCR	A close correlation was found between low miR-597 expression with positive lymph node metastasis advanced TNM stage poorer tumor differentiation, unfavorable OS and higher expression levels of miR-597. miR-597 can be an independent prognostic indicator of BC
[97]	miR-29b	121 BC and 56 benign breast tissue specimens	qRT-PCR	Increased levels of miR-29b had a significantly longer disease-free survival and a lower risk to relapse
[98]	miR-301	380 BC samples	TCGA	Higher expression of miR-301a in BC cases is correlated with reduction of 5-year DFS and OS compared to BC with low levels of miR-301a expression.
[99]	MALAT1	80 BC cases compared to 80 controls.	qRT-PCR	A positive correlation was observed between MALAT1 expression with lymph node status, ER status, tumor stage, and histological grade indicating its possible prognostic value.
[100]	lncRNA00544	373 primary BC samples of 49 BC tissues and pair-matched metastatic axillary nodes	qRT-PCR	Elevated expression of lncRNA00544 was correlated with poor disease-free survival. lncRNA00544 can represent a novel predictive and prognostic biomarker in luminal BC patients.
[117]	miR-9 and miR-155	190 TNBC	qRT-PCR	Increased miR-9 levels showed significant association with poor PFS and distant metastasis–free survival (DMFS) in TNBC, whereas high level of miR-155 expression was associated with better DMFS miR-9 and miR-155 can be prognostic biomarkers in TNBCs.
[118]	miR-629-3p	669 patients without de novo stage IV TNBC	qRT-PCR and IHC	miR-629-3p was correlated with poor OS and DFS in the validation set, but it failed to show significance after multivariate analysis.
[119]	miR-101	781 patients with BC	TCGA	Low levels of miR-101-2 expression might represent a diagnostic) marker, whereas the miR-101-1 was a prognostic marker. There was a close correlation between ER, PR, and HER2, while miR-101-2 was correlated with the tumor (T), lymph node (N), and metastasis (M) stages of BC.
[120]	LincIN	781 BC patients	TCGA	Overexpression of LincIN was associated with BC aggressiveness and shorter OS. Ablation of LincIN showed inhibition of tumor cell migration and invasion in vitro and diminished lung metastasis in a mouse tail vein injection model.
[121]	SPRY4-IT1	110 BC	qRT-PCR	Increased expression of Z38 was found in BC compared to controls, advanced TNM stage, presence of lymph node metastasis and unfavorable OS.
[122]	10 miRNAs, including (miR-7,-21,-29a,-29b,-34a,-125b,-155,-200c,-340,-451)	64 BC patients	PCR	The patients with miR-7low or miR-340 high profile might not have complete response.
[123]	miR-199b-5p	19 BC samples	qRT-PCR	Low expression of MiR-199b-5p showed close association with advanced TNM stage, positive lymph node metastasis and poor OS miR-199b-5p might be a possible marker for BC.
[124]	MALAT1	33 pairs of primary non-metastatic BC and their matched adjacent normal tissues 204 BC tissues	TCGA	Up-regulation of MALAT1 was associated with poor RFS in tamoxifen-treated ER-positive BC patients, which might present as a candidate biomarker to predict endocrine treatment sensitivity.
[125]	12 circulating miRNAs in serum of inflammatory and non-inflammatory BC	1014 BC patients	TCGA	Overexpression of miR-335 in premenopausal non-inflammatory BC patients, whereas miR-24 was significantly upregulated in non-inflammatory BC with postmenopausal status.
[126]	MALAT1	446 unilateral invasive primary BC	RT-PCR	The authors reported a complex expression pattern of various MALAT1 transcript variants in BC cases and the prognostic and predictive role of MALAT1 should be considered conservatively.
[127]	Hsa-miR-375	115 patients (30 relapses versus 85 controls)	qRT-PCR	Positive association between the levels of has-miR-375 with local relapshashsa-miR-375 can distinguish between relapse and control groups
[128]	miR-520g	86 cases with BC	qRT-PCR	Higher levels of miR-520g were found in BC patients with lymph node metastatic and low differentiation degree grade mammary gland invasion and low expression of p53. miR-520g might be a potential prognostic factor in BC.
[129]	miR-200a, miR-200b, miR-200c, miR-210, miR-215 and miR-486-5p	Primary BC with metastasis (M1, *n* = 67) at diagnosis/blood collection, and patients without metastasis at diagnosis (M0, *n* = 265) plasma miRNAs of 40 MBC patients 237 MBC patients	TaqMan low density arrays	A significant correlation was found between miR-200a, miR-200b, miR-200c, miR-210, miR-215, and miR-486-5p with metastasis development before clinical manifestation of BC.
[130]	CCAT1	92 pairs of BC cancer tissues and adjacent normal tissues	qRT-PCR	Significant correlation between CCAT1 with poor differentiation grade, advanced TNM stage, presence of lymph node metastases, and shorter OS and PFS. CCAT1 could be a possible prognostic marker for BC progression.
[131]	miR-124	133 BC patients	qRT-PCR	This group showed that miR-124 can be an indicator of tumor progression and poor prognosis in BC cases.
[132]	lncRNA microarray data from 164 primary breast tumors from adjuvant naïve patients	82 patients cases with detectable distant metastasis were compared to 82 patients with no metastases.	Microarray analysis	That lncRNA profiles could distinguish metastatic patients from non-metastatic patients with sensitivities above 90% and specificities of 64–65%.
[133]	HOTAIR	133 BC cases	TCGA	HOTAIR might be an indicator of lymphatic metastases rather than hematogenous metastases in ER—BC.
[134]	miR-21	549 cases (326 with breast cancer, 223 without breast cancer)	(RQ)-PCR	Increased expression ofmiR-21 was reported in tissues and serum of BC patients versus healthy control groups in the Chinese population and it can be an indicator of recurrence and disease-free survival
[135]	miR-106b	Plasma samples of 173 patients with primary BC and a set of 50 women with fibroadenoma	qRT-PCR and in situ hybridization.	Increased levels of miR-106b were correlated with higher Ki67 expression, lymph node metastasis, shorter PFS, and OS. miR-106b might be a high risk of recurrence of BC.
[136]	miR34a	Three independent primary BC cohorts (Cohort 1 with 461, Cohort 2 with 279 and Cohort 3 with 795 patients)	quantitative in situ hybridisation assay (qISH)	Loss of miR34a can distinguish patients with poor PSF among node-negative patients, but not in the node-positive population. Loss of miR34a might be an indicator of a subgroup of BC patients with unfavorable disease-specific survival
[137]	miR-21, miR-210, and miR-373	Serum of 127 HER2-postive BC patients before and after neoadjuvant therapy and 19 healthy controls	TaqMan MicroRNA assays	Close association between neoadjuvant therapy and the serum levels of miR-21, miR-210, and miR-373 in BC cases with a prognostic value for miR-21.
[138]	miR-27b-3p, miR-107, and miR-103a-3p	99 TNBC patients including a training set of 58 patients with invasive ductal TNBC further validated in a separate set of 41 TNBC patients	qRT-PCR	Expressions of miR-27b-3p, miR-107, and miR-103a-3p were significantly up-regulated in the metastatic group versus the disease-free. Lymph node status and miR-27b-3p were independent predictors of poor prognosis.
[139]	miR-10b	101 paired tumor and normal specimens	qRT-PCR	Adding miR-10b RERs to the prognostic factors used in clinical routine could improve the prediction abilities for overall mortality as well as progression in BC patients.
[140]	miR-630	all breast tissue (*n* = 56) and HER2+ breast tissue (*n* = 6)	qRT-PCR	Induction of miR-630 into cells with innate- or acquired- resistance to HER-drugs significantly restored the efficacy of lapatinib, neratinib and afatinib
[141]	miR-149, miR-10a, miR-20b, miR-30a-3p, and miR-342-5p,	71 primary BC	Microarray and RT-qPCR	These five 5-miRNA signatures determined a high-risk group of patients with shorter relapse-free survival as well as non-relapsing versus early-relapsing patients. possible prognostic value to identify patients with metastasis development after primary breast surgery.
[142]	miR-155, miR-493, miR-30e and miR-27a	160 TNBC	First: miRNA expression profiling with GEO and TCGA Second: TaqMan^®^ qRT-PCR assay	TNBC subclassification based on the 5 IHC markers and on the miR-155, miR-493, miR-30e, miR-27a expression levels are a powerful diagnostic approach.
[143]	miR-26a, miR-26b, miR-203, miR-421, miR-664, miR-576-5p and miR-18a	52 BC and 3 normal breast samples	qRT-PCR	Increased expression of miR-421 was detected in 36.5% of cases which exhibit lower ATM transcript levels. It is clear that ATM protein expression might represent an independent prognostic marker in sporadic BC.
[144]	HOTAIR	164 primary BC without adjuvant therapy	microarray	HOTAIR expression may provide an independent biomarker for the prediction of the risk of metastasis in ER + BC patients.
[145]	CCAT2	997 primary BC	qRT-PCR and ISH	CCAT2 up-regulates cell migration and down-regulates chemosensitivity to 5’FU in a rs6983267-independent manner.
[146]	RAD21	28 BRCA1, 27 BRCA2, and 39 BRCAX	IHC	RAD21 is a potential predictive and prognostic biomarker in familial breast cancers.
[147]	miR-205	40 FFPE archival BC	in situ hybridization analysisof micro-RNA expression in arrays	Expression of miR-205 is associated with tumors of ductal morphology and thus this molecule can be considered as a prognostic marker within these tumours.
[148]	miR-210	56 systemically untreated BC patients	miRNA microarray hybridization qRT-PCR	The effects of MiR-210 were analyzed on the BC cells, including MCF7 and MDA-MB-231. MiR-210 expression showed that this molecule was involved in cell proliferation, migration and invasion.
[149]	miR-106b	103 lymph node negative BC	miRCURY LNA Array	Also demonstrated the presence of several microRNAs, including miR532-5p, miR-500, miR362-5p, and miR502-3p, located at Xp11.23 in cancers with a triple-negative signature, and the increased expression of several miR-17 cluster members in ER− tumors.
[150]	Dicer expression	104 BC patients	qRT-PCR	A close correlation was reported between Dicer protein expression and hormone receptor status and subtypes in BC (Dicer expression might be an indicator of distant metastases in BC cases.
[151]	8 mRNAs and 2 lncRNAs	In the training cohort, a total of 198 frozen tissues from 165 consecutive TNBC patients (including 33 pairs of tumor and adjacent normal tissues) 266 frozen TNBC samples and 33 adjacent normal breast tissue	First: GEO Second: validation with qRT-PCR	Tumor-specific mRNAs and lncRNAs were identified and correlated with patients’ recurrence-free survival (RFS).
[152]	EPB41L4A-AS2	250 BC tissues and 50 adjacent normal tissues	qRT-PCR	Induction of EPB41L4A-AS2 expression inhibited breast tumor cell proliferation. It can be concluded that evaluation of this long non-coding RNA might provide a possible prognostic biomarker in the clinical management of BC.
[153]	miR-29c and miR-101	2178 BC	GEO, EGA, TCGA	The authors demonstrated that miR-29c and miR-101 might have prognostic value in BC patients.
[154]	miR-205	30 BC patients	qRT-PCR	miR-205 may be valuable for prediction of the TAC regimen as well as a possible therapeutic target in BC treatment.
[155]	miR-7, miR-22, miR-21, miR-30c, miR-181a, miR-181c, miR-125b, miR-200a, miR-135b, and miR-200c	818 BC patients	qRT-PCR	The 10-miRNAs were a good prognostic biomarker to predict distant relapse free survival (DRFS) in BC.
[156]	12 differentially expressed lncRNAs	473 BC patients	GEO	This lncRNAs was closely associated with tumor recurrence of BC from discovery cohort, which was capable to classify patints into high-risk and low-risk with recurrence-free survival that was significantly
[157]	Holistic IDFO approach of prioritization of cancers	5 human cancers, including breast cancer, in 3198 samples	TCGA	lncRNAs closely associated with tumor recurrence of BC from discovery cohort, which was capable to classify patients into high-risk and low-risk with recurrence-free survival that was significantly different
[158]	14 miRNAs	Training (*n* = 596) and Testing set (*n* = 319)	TCGA	Patients could be characterized as high and low score according to the risk scores calculated for each miRNA. The signature could be used as prognostic marker in ER+ BC.
[159]	Drosha and Dicer	245 patients receiving adjuvant anthracycline-based chemotherapy	qRT-PCR	Concurrent down-regulation of Drosha and dicer in 15% of cases and a significant association with both high grade and ki-67 index. No significant association between the down-regulation of Drosha and/or Dicer and outcomes.
